# The Cohesive Interactions in Phenylimidazoles

**DOI:** 10.1021/acs.jpca.4c01589

**Published:** 2024-05-30

**Authors:** José C. S. Costa, Ana I. M. C. Lobo Ferreira, Carlos F. R. A. C. Lima, Luís M. N. B. F. Santos

**Affiliations:** CIQUP, Institute of Molecular Sciences (IMS), Department of Chemistry and Biochemistry, Faculty of Science, University of Porto, Rua do Campo Alegre s/n, Porto P4169-007, Portugal

## Abstract

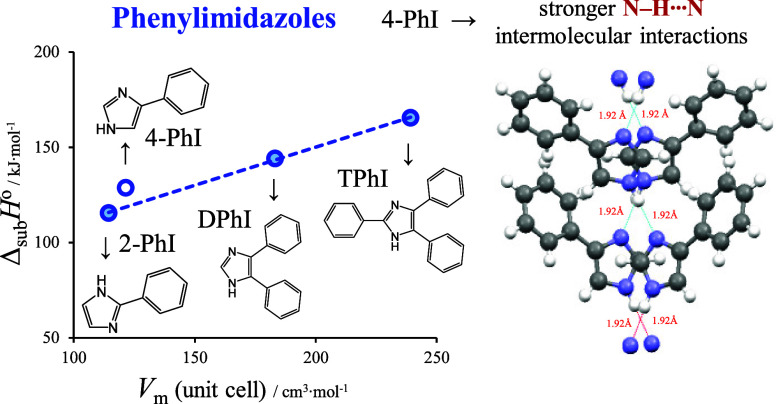

This work presents a comprehensive study exploring the thermodynamics of the solid phase of a series of phenylimidazoles, encompassing experimental measurements of heat capacity, volatility, and thermal behavior. The influence of successive phenyl group insertions on the imidazole ring on thermodynamic properties and supramolecular behavior was thoroughly examined through the evaluation of 2-phenylimidazole (2-PhI), 4-phenylimidazole (4-PhI), 4,5-diphenylimidazole (4,5-DPhI), and 2,4,5-triphenylimidazole (2,4,5-TPhI). Structural correlations between molecular structure and thermodynamic properties were established. Furthermore, the investigation employed UV–vis spectroscopy and quantum chemical calculations. Additive effects arising from the introduction of phenyl groups were found through the analysis of the solid–liquid and solid–gas equilibria, as well as heat capacities. A good correlation emerged between the thermodynamic properties of sublimation and the molar volume of the unit cell, evident across 2-PhI, 4,5-DPhI, and 2,4,5-TPhI. In contrast to its isomer 2-PhI, 4-PhI exhibited greater cohesive energy due to the stronger N–H···N intermolecular interactions, leading to the disruption of coplanar geometry in the 4-PhI molecules. The observed higher entropies of phase transition (fusion and sublimation) are consistent with the higher structural order observed in the crystalline lattice of 4-PhI.

## 1 Introduction

Imidazole derivatives form a distinct category of organic compounds characterized by the incorporation of an imidazole ring—a five-membered heterocyclic structure containing two nitrogen atoms and three carbon atoms. Phenylimidazoles stand out within this class due to the addition of phenyl groups, imparting unique properties and applications to these compounds. Demonstrating remarkable versatility across various scientific disciplines, phenylimidazoles exhibit noteworthy biological activities. Certain derivatives showcase antimicrobial, antifungal, and anti-inflammatory properties, positioning them as promising candidates for drug development.^1−3^ The significance of phenylimidazoles extends to the field of materials science, where they play a pivotal role in synthesizing polymers and coordination compounds employed in various applications such as sensors, catalysis, and optoelectronics.^4−6^ The unique structural characteristics and versatile reactivity of phenylimidazoles render them of paramount importance in the fabrication of functional materials.^7−9^ Moreover, imidazole derivatives play a crucial role in the realm of ionic liquids, where the imidazole ring acts as a fundamental building block to finely tune desired properties.^10−12^

**Figure 1:**
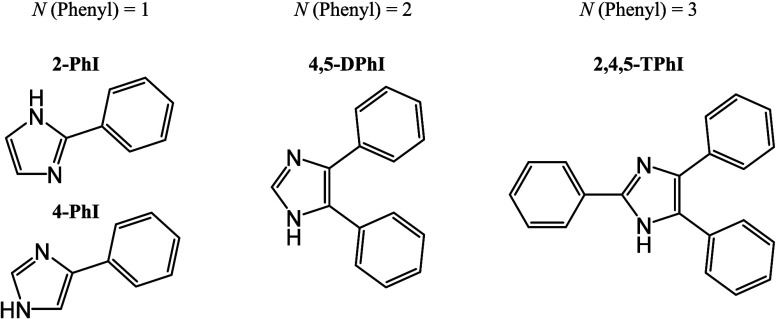
Molecular structures of the studied materials and their corresponding abbreviations: 2-phenylimidazole (2-PhI), 4-phenylimidazole (4-PhI), 4,5-diphenylimidazole (4,5-DPhI), and 2,4,5-triphenylimidazole (2,4,5-TPhI).

This investigation is dedicated to the study of phenylimidazoles characterized by varying numbers of phenyl groups attached to the central imidazole ring, as elucidated in Figure 1. The compounds under scrutiny encompass the isomers 2-phenylimidazole (2-PhI) and 4-phenylimidazole (4-PhI), 4,5-diphenylimidazole (4,5-DPhI), and 2,4,5-triphenylimidazole (2,4,5-TPhI). 2-PhI and 4-PhI are used in the synthesis of metal complexes and serve as key intermediates in the production of agrochemicals, pharmaceuticals, and specialty products. Additionally, they are employed as curing agents for epoxy resins and polyurethane.^13−19^

4,5-DPhI has found widespread applications in the spectrophotometric determination of metal ions, serving as a chromogenic reagent.^20,21^ Moreover, derivatives of 4,5-DPhI have been documented to demonstrate a diverse array of pharmacological properties.^22,23^ 2,4,5-TPhI (also known as Lophine) is an attractive fluorescence and chemiluminescence poly-substituted imidazole compound.^24^ Derivatives of 2,4,5-TPhI are widely used in textiles, photographic materials, electroluminescent materials, and optical applications.^25−29^

This research aims to provide a meticulous structural and thermodynamic characterization of each phenylimidazole, yielding useful knowledge for applications spanning process optimization, material characterization, and considerations related to energy efficiency.^25−33^ The crystalline structures of 2-PhI, 4-PhI, 4,5-DPhI, and 2,4,5-TPhI have been well documented in the literature.^24,34−38^ However, a comprehensive understanding of the thermodynamic properties of these organic compounds remains incomplete. In this context, the principal objective is to evaluate the influence of successive phenyl group introductions on the thermodynamic properties and supramolecular characteristics of these materials. The investigation specifically focuses on phase equilibrium thermodynamics and establishes correlations between molecular structure and thermodynamic properties. The thermodynamic evaluation of the solid phase of each phenylimidazole includes experimental measurements of heat capacity, volatility, and thermodynamic properties of phase transition. In addition, experimental data on thermodynamic properties are complemented by quantum chemical calculations and UV–vis spectroscopy. By presenting novel insights into the physical chemistry of phenylimidazoles, this study advances foundational knowledge on this class of compounds and contributes to the rational design of innovative materials.

## 2 Experimental Section

### 2.1 Materials

The compounds under study, 2-phenylimidazole (2-PhI; CAS number 670–96–2; mass fraction 0.992), 4-phenylimidazole (4-PhI; CAS number 670–95–1; mass fraction 0.995), 4,5-diphenylimidazole (4,5-DPhI; CAS number 668–94–0; mass fraction 0.999), and 2,4,5-triphenylimidazole (2,4,5-DPhI; CAS number 484–47–9, mass fraction 0.999), were commercially purchased from Sigma-Aldrich/Merck. For enhanced purity, 2-PhI, 4-PhI, 4,5-DPhI, and 2,4,5-TPhI underwent sublimation under reduced pressure (<10 Pa) at temperatures of *T* = 403.2 K, *T* = 403.2 K, *T* = 453.2 K, and *T* = 483.2 K, respectively. The final purity of each compound (mass fraction > 0.999) was confirmed through gas–liquid chromatography using an Agilent chromatograph, model 4890D, featuring an HP-5 column and a flame ionization detector (FID).

### 2.2 High-Precision Heat Capacity Drop Calorimetry

The heat capacities in the solid phase at *θ* = 298.15 K were measured for each compound using a high-precision heat capacity drop calorimeter.^39^ Sample masses ranging from 0.200 to 0.500 g were used. The calibration of the calorimeter was performed with sapphire (α-Al_2_O_3_, NIST-RM 720), employing the standard molar heat capacity, *C*_*p*,m_^°^, at 298.15 K, as documented in the literature: *C*_*p*,m_^°^(α-Al_2_O_3_, 298.15 K) = (79.03 ± 0.08) J·K^–1^·mol^–1^.^40^ The calibration constant was found to be *ε* = (6.6683 ± 0.0174) W·V^–1^. A comprehensive description of the calorimeter, along with assessments of its reproducibility and precision for measuring *C*_*p*,m_^°^ of condensed samples, can be found in previous publications.^39,41,42^ The quoted uncertainties were considered twice the standard deviation of the mean and included calibration uncertainty.

### 2.3 Differential Scanning Calorimetry

Melting temperatures and enthalpies of fusion for each compound were determined using a power compensation differential scanning calorimeter, specifically the PerkinElmer model Pyris Diamond DSC. Hermetically sealed aluminum crucibles were employed, and the DSC experiments were conducted on phenylimidazole samples with a weight range between 5 and 10 mg. The scanning rate utilized was 5 K·min^–1^, and a constant flow of nitrogen (20 mL·min^–1^) was maintained during each experiment. The DSC’s temperature and heat flux scales were calibrated through the measurement of the temperatures and enthalpies of fusion of various reference materials. These included benzoic acid, *o*-terphenyl, naphthalene, anthracene, 1,3,5-triphenylbenzene, perylene, 1-hexanol, 1-heptanol, diphenylether, and 1,3-difluorobezene.^40,43−46^

### 2.4 Knudsen Effusion with Quartz Crystal Microbalance

The Knudsen effusion methodology is widely used for evaluating the volatility of organic materials at pressures below 1 Pa.^47−51^ In our investigation, we determined the vapor pressures of the solid phase of each phenylimidazole as a function of temperature using a combined Knudsen/quartz crystal effusion apparatus.^49^ Specifically, vapor pressure measurements for 2-PhI and 4-PhI, due to their relatively high volatility, were conducted using the gravimetric Knudsen effusion methodology. Vapor pressure measurements for 4,5-DPhI and 2,4,5-TPhI were carried out using the combined Knudsen/quartz crystal effusion methodology. The vapor pressures were determined within the following temperature intervals: 2-PhI (355.3–374.9 K, 0.156–1.18 Pa); 4-PhI (362.1–382.8 K, 0.116–1.18 Pa); 4,5-DPhI (408.0–434.8 K, 0.0804–1.05 Pa); 2,4,5-TPhI (444.0–471.1 K, 0.0856–1.03 Pa). The raw data, along with supplementary details regarding the methodology, are provided in the Supporting Information.

### 2.5 UV–vis Spectroscopy

UV–vis spectra of 2-PhI, 4-PhI, 4,5-DPhI, and 2,4,5-TPhI in CH_2_Cl_2_ solutions (concentrations of approximately 10^–5^ mol·dm^–3^) were recorded in the 200–800 nm range. The measurements were conducted using an Agilent 8453 diode array UV–vis spectrometer at a temperature of 298.1 K. A quartz cell with a path length of 10.00 mm was utilized, and temperature control was achieved through a Julabo F25 HP refrigerated circulator.

### 2.6 Computational Details

Quantum chemical calculations were performed using the Gaussian 09 software package.^52^ Full geometry optimizations and frequency calculations, without symmetry restrictions, were conducted for 2-PhI, 4-PhI, 4,5-DPhI, and 2,4,5-TPhI at the M06-2X/6-311++G(d,p) level of theory. No imaginary frequencies were found, confirming that the optimized structures correspond to true minima. These calculations were used to determine the gaseous phase heat capacities for all species at 298.15 K, using the recommended frequency scaling factor of 0.9567,^53^ and evaluated gas phase molecular geometries and energetics. In addition, TD-DFT at the M06-2X/6-311++G(d,p) level of theory was employed to simulate the UV–vis spectra of the phenylimidazoles studied by calculating the excitation energies and oscillator strengths considering 8 to 16 excited states.^54−57^ The UV–vis absorption spectra were simulated by assuming a Gaussian band shape with a peak width of 0.4 eV. The polarizable continuum model (PCM) was used to simulate the solvent effect (CH_2_Cl_2_).^58^ Detailed computational results are provided in the Supporting Information.

## 3 Results and Discussion

### 3.1 Heat Capacities

**Figure 2:**
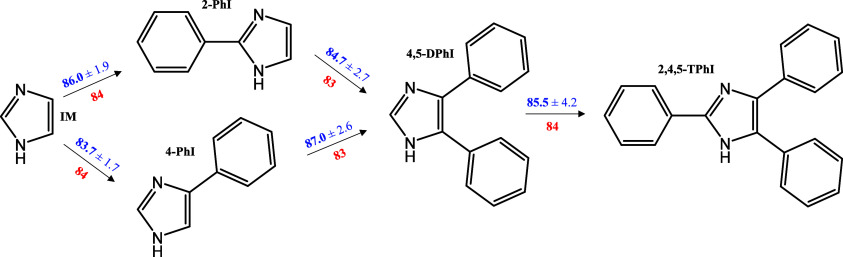
Additive scheme for *C*_*p*,m_^°^ (values in J·K^–1^·mol^–1^ at 298.15 K) illustrating the incremental changes in this property upon the successive introduction of phenyl groups. The blue values (experimental results) above the arrow denote the increment for *C*_*p*,m_^°^ in the solid phase, while the red values (theoretical values obtained at M06-2X/6-311++G(d,p) level of theory) below the arrow represent the increment for *C*_*p*,m_^°^ in the gas phase.

Table 1 presents the heat capacity values (at *θ* = 298.15 K) in the solid phase, including molar (*C*_*p*,m_^°^(s)) and specific (*c*_*p*,_^°^(s)) values, as well as in the gas phase (*C*_*p*,m_^°^(g)), for 2-PhI, 4-PhI, 2,4-DPhI, and 2,4,5-TPhI. Literature values for imidazole (IM) are included for comparative analysis.^59−61^ The derived Δ_sub_*C*_*p*,m_^°^ values, calculated as *C*_*p*,m_^°^(g) – *C*_*p*,m_^°^(s), are also listed. Experimental measurements were employed for *C*_*p*,m_^°^(s), while *C*_*p*,m_^°^(g) was determined through quantum chemical calculations. Detailed results regarding the experimental determination of *C*_*p*,m_^°^(s) are provided in the Supporting Information. The successive introduction of phenyl groups resulted in an additive increment in the heat capacity for both solid and gas phases. To enhance visualization, Figure 2 presents an additive scheme illustrating the incremental changes in *C*_*p*,m_^°^(s) and *C*_*p*,m_^°^(g) upon the successive introduction of phenyl groups.

**Table 1:** Values of the Standard Molar Heat Capacity in the Solid Phase (*C*_*p*,m_^°^(s)) and Specific Heat Capacity in the Solid Phase (*c*_*p*_^°^ (s)), as well as the Standard Molar Heat Capacity in the Gas Phase (*C*_*p*,m_^°^(g)) for Imidazole (IM), 2-Phenylimidazole (2-PhI), 4-Phenylimidazole (4-PhI), 4,5-Diphenylimidazole (4,5-DPhI), and 2,4,5-Triphenylimidazole (2,4,5-TPhI)^a^

compound	*C*_*p*,m_^°^(s) / J·K^–1^·mol^–1^	ref	*c*_*p*_°(s) / J·K^–1^·g^–1^	*C*_*p*,m_^°^(g) / J·K^–1^·mol^–1^	Δ_sub_*C*_*p*,m_^°^ / J·K^–1^·mol^–1^
IM	84.6	ref (59)	1.24		
	82.4	ref (60)	1.21	66 ± 5	–18 ± 5
	83.9	ref (61)	1.23		
	83.6 ± 1.1	average value			
2-PhI	169.6 ± 1.5	this work	1.18 ± 0.01	150 ± 5	–19 ± 5
4-PhI	167.3 ± 1.3	this work	1.16 ± 0.01	150 ± 5	–17 ± 5
4,5-DPhI	254.3 ± 2.2	this work	1.15 ± 0.01	233 ± 5	–21 ± 6
2,4,5-TPhI	339.8 ± 3.6	this work	1.15 ± 0.01	317 ± 5	–23 ± 6

a The uncertainty reported for (s) is twice the standard deviation of the mean and the calibration uncertainty is included. The uncertainty of (g) was considered to be ±5 J·K^–1^·mol^–1^. The derived Δ_sub_*C*_*p*,m_^°^ values calculated as *C*_*p*,m_^°^(g) – *C*_*p*,m_^°^(s) are also listed. All parameters are referenced at *θ* = 298.15 K.

The increment in *C*_*p*,m_^°^(g) through successive introductions of a phenyl group in the imidazole central ring was found to be (84 ± 1) J·K^–1^·mol^–1^. Similarly, for *C*_*p*,m_^°^(s), the increments are very comparable within the experimental uncertainties. The average of all the increments reveals that the insertion of a phenyl group results in a rise in *C*_*p*,m_^°^(s), with a determined increment of (85 ± 1) J·K^–1^·mol^–1^. This value does not significantly differ from the increment in *C*_*p*,m_^°^(s) observed in linear oligophenyls. For instance, an increment of 81.2 J·K^–1^·mol^–1^ is observed between biphenyl and *p*-terphenyl, and an increment of 83.9 J·K^–1^·mol^–1^ is observed between *p*-terphenyl and *p*-quaterphenyl.^39,62,63^ According to our results, 4,5-DPhI and 2,4,5-TPhI exhibit identical *c*_*p*_^°^(s) values (1.15 J·K^–1^·g^–1^), while slightly larger values were obtained for the isomers 2-PhI and 4-PhI. The similarity in *C*_*p*,m_^°^(s) and *C*_*p*,m_^°^(g) increments illustrate the similar effect of successive addition of phenyl groups to imidazole, considering the contributions of intermolecular potential and extra molecular vibrations for the heat capacity.

### 3.2 Thermodynamic Properties of Fusion

The thermodynamic properties related to the fusion process of 2-PhI, 4-PhI, 4,5-DPhI, and 2,4,5-TPhI are presented in Table 2. The melting temperatures (*T*_m_), as well as the molar enthalpies of fusion (Δ_fus_*H*_m_^°^) and molar entropies of fusion (Δ_fus_*S*_m_^°^) at *T*_m_ are provided. Literature values for IM are included for comparison.^59,64−67^ The DSC thermograms are provided in the Supporting Information. The samples underwent double cycles of heating and cooling. In the initial heating scan, 2-PhI, 4-PhI, 4,5-DPhI, and 2,4,5-TPhI exhibited the characteristic endothermic peak indicative of the melting process. The *T*_m_ values obtained are as follows: *T*_m_ = (421.1 ± 0.9) K for 2-PhI; *T*_m_ = (414.4 ± 0.5) K for 4-PhI; *T*_m_ = (506.6 ± 0.9) K for 4,5-DPhI; *T*_m_ = (549.4 ± 0.9) K for 2,4,5-TPhI. During the cooling cycle, 2-PhI, 4,5-DPhI, and 2,4,5-TPhI exhibited complete crystallization, and no glassy state was detected for these phenylimidazoles. However, 4-PhI did not show complete crystallization. The second heating cycle enabled the determination of the glass transition temperature (*T*_g_) and the cold crystallization temperature (*T*_cc_) of 4-PhI as follows: *T*_g_ = (285.6 ± 1.0) K; *T*_cc_ = (323.2 ± 1.4) K. While these transitions are detectable, they were found to be very weak. For the isomer 4-PhI, two polymorphic modifications (*t*-4PhI and *m*-4PhI) have been reported in the literature.^69^ These polymorphs exhibit thermodynamic properties of fusion that are similar within the experimental uncertainty. Our compound corresponds to *m*-4PhI, as it has been reported that for *t*-4PhI, a visible thermal glass transition occurs, whereas, for *m*-4PhI, the glass transition is reported to be very weak.^70^

**Table 2:** Experimental Results and Literature Data for the Thermal Properties of Imidazole (IM), 2-Phenylimidazole (2-PhI), 4-Phenylimidazole (4-PhI), 4,5-Diphenylimidazole (4,5-DPhI), and 2,4,5-Triphenylimidazole (2,4,5-TPhI): Melting Temperature (*T*_m_), Standard Molar Enthalpy (Δ_fus_*H*_m_^°^), and Molar Entropy (Δ_fus_*S*_m_^°^) of Fusion at the Melting Temperature^a^

	*T*_m_	Δ_fus_*H*_m_^°^(*T*_m_)	Δ_fus_*S*_m_^°^(*T*_m_)		Δ_fus_*H*_m_^°^(θ)	Δ_fus_*S*_m_^°^(θ)	Δ_fus_*G*_m_^°^(θ)
compound	K	kJ·mol^–1^	J·K^–1^·mol^–1^	ref	kJ·mol^–1^	J·K^–1^·mol^–1^	kJ·mol^–1^
IM	363.7	11.6	31.9	ref (59)			
	361.9	12.8	35.4	ref (64)			
	359.3	13.1	36.5	ref (65)	8.9 ± 1.3	23.5 ± 3.9	1.9 ± 1.7
	363.7	12.5	34.4	ref (66)			
	362.3	11.8	32.6	ref (67)			
2-PhI	421.1 ± 0.9	18.1 ± 1.0	43.0 ± 2.4	this work	11.4 ± 2.5	24.2 ± 6.9	4.2 ± 3.2
	420.0	17.8	42.4	ref (68)			
	422.9	17.9	42.3	ref (69)			
	420.1	15.3	36.5	ref (70)			
4-PhI	414.4 ± 0.5	20.6 ± 1.0	49.7 ± 2.4	this work	14.3 ± 2.3	31.8 ± 6.6	4.8 ± 3.0
	418.9 (*t*-4PhI)	21.3 (*t*-4PhI)	50.9 (*t*-4PhI)	ref (70)			
	417.2 (*m*-4PhI)	20.3 (*m*-4PhI)	48.7 (*m*-4PhI)	ref (70)			
4,5-DPhI	506.6 ± 0.9	35.4 ± 1.0	69.8 ± 2.0	this work	24.1 ± 4.2	41.0 ± 10.6	11.8 ± 5.2
	505.0	32.3	64.0	ref (68)			
	504.9	34.2	67.7	ref (69)			
2,4,5-TPhI	549.4 ± 0.9	46.0 ± 1.0	83.7 ± 1.8	this work	32.3 ± 5.0	50.4 ± 12.2	17.3 ± 6.2
	547.8	37.3	68.1	ref (68)			
	550.8	35.2	63.9	ref (71)			

a The reported uncertainties for *T*_m_ and Δ_fus_*H*_m_^°^(*T*_m_) are twice the standard deviation of the mean and include the calibration uncertainty. For the other parameters, the combined uncertainty was derived. Derived thermodynamic properties of fusion at *θ* = 298.15 K are also listed: hypothetical molar enthalpy of fusion (Δ_fus_*H*_m_^°^(*θ*)), hypothetical molar entropy of fusion (Δ_fus_*S*_m_^°^(*θ*)), and hypothetical molar Gibbs energy of fusion (Δ_fus_*G*_m_^°^(*θ*)). The thermodynamic properties of fusion at *θ* = 298.15 K were determined from experimental data obtained at *T*_m_ by adjusting for heat capacity differences between the liquid and solid phases. Values presented for IM were derived from an average of the literature results. Values presented for 2-PhI, 4-PhI, 4,5-DPhI, and 2,4,5-TPhI were derived from the experimental data obtained in this work.

The values of *T*_m_, Δ_fus_*H*_m_^°^, and Δ_fus_*S*_m_^°^ obtained for the isomers 2-PhI and 4-PhI are in reasonable agreement with the values reported in the literature.^67−69^ The higher *T*_m_ obtained for 2-PhI was found to be entropically driven. Considering that *T*_m_ results from the Δ_fus_*H*_m_^°^/Δ_fus_*S*_m_^°^ ratio, higher values of Δ_fus_*H*_m_^°^ and/or lower values of Δ_fus_*S*_m_^°^ contribute to increase *T*_m_. According to the experimental data, the lower Δ_fus_*S*_m_^°^ value is the preponderant factor for the higher *T*_m_ of 2-PhI. The values determined for 4,5-DPhI are also consistent with the available literature data.^68,69^ Nevertheless, our findings for 2,4,5-TPhI reveal disparities when juxtaposed with values reported by other researchers.^68,71^ While our determined *T*_m_ aligns closely, the observed Δ_fus_*H*_m_^°^ is significantly larger than the values documented in the existing literature. To facilitate an accurate comparison of the thermodynamic properties across all compounds, we opt for using the same reference temperature and evaluate the increase in both Δ_fus_*H*_m_^°^ and Δ_fus_*S*_m_^°^ with the successive introduction of phenyl groups in the central imidazole ring. For this, we have calculated the thermodynamic properties at *θ* = 298.15 K. These properties were derived from experimental data obtained at *T*_m_, accounting for heat capacity differences between the liquid and solid phases. Detailed analyses are presented in the Supporting Information. Both Δ_fus_*H*_m_^°^(*θ*) and Δ_fus_*S*_m_^°^(*θ*) are significantly larger for 4-PhI compared to its corresponding isomer, 2-PhI. When comparing the isomers PhI with the congeners 4,5-DPhI and 2,4,5-TPhI, the successive insertion of an additional phenyl group leads to an expected increase in the value of Δ_fus_*H*_m_^°^(θ).

### 3.3 Thermodynamic Properties of Sublimation and Vaporization

**Figure 3:**
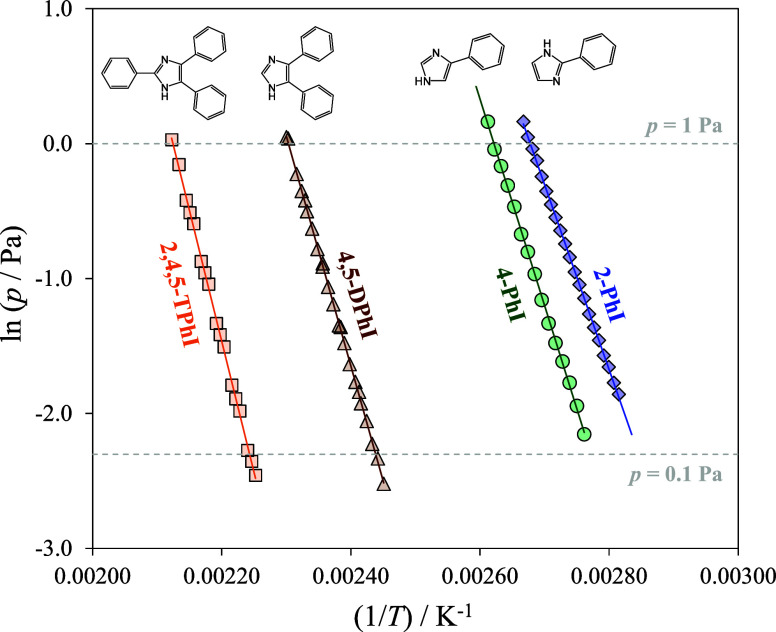
Experimental plots of ln(*p*) versus 1/*T* for 2-PhI (diamonds), 4-PhI (circles), 4,5-DPhI (triangles), and 2,4,5-TPhI (squares).

Figure 3 displays ln(*p*/Pa) = *f*[1/*T* (K^–1^)] plots derived from Knudsen effusion experiments conducted on high-purity samples of 2-PhI, 4-PhI, 4,5-DPhI, and 2,4,5-TPhI. Table 3 details the results of fitting the sublimation data to the linear Clausius–Clapeyron equation for the compounds studied including the mean temperature of the effusion experiments (⟨*T*⟩), the respective equilibrium vapor pressure (*p*⟨*T*⟩), and the derived molar enthalpies of sublimation (Δ_sub_*H*_m_) and molar entropies of sublimation (Δ_sub_*S*_m_), at ⟨*T*⟩ and *p*⟨*T*⟩. In Table 4, standard molar thermodynamic parameters of sublimation at θ = 298.15 K are compiled. The values for IM, derived from available literature data, are provided for comparison. By considering the thermodynamic properties of both fusion and sublimation, insights into the thermodynamic properties of vaporization can be inferred. The corresponding values are also included. Δ_sub_*H*_m_^°^ (*θ*) and Δ_sub_*S*_m_^°^ (*θ*) were determined from experimental data obtained at ⟨*T*⟩, with heat capacity corrections accounting for differences between the gas and solid phases (Table 1). A comprehensive overview of sublimation results and data analysis, incorporating heat capacity corrections, is available in the Supporting Information. It is noteworthy to mention that to the best of our knowledge, there is no literature data on the volatility of these compounds, underscoring the novelty of our experimental results.

**Table 3:** Experimental Sublimation Results Obtained for 2-Phenylimidazole (2-PhI), 4-Phenylimidazole (4-PhI), 4,5-Diphenylimidazole (4,5-DPhI), and 2,4,5-Triphenylimidazole (2,4,5-TPhI): Parameters (*a* and *b*) of the Clausius–Clapeyron Equation (ln(*p*/Pa) = *a* – *b*/(*T*/K)), the Mean Temperature of the Effusion Experiments (⟨*T*⟩), and the Respective Equilibrium Vapor Pressure (*p*⟨*T*⟩);^a^ the Molar Enthalpies of Sublimation (Δ_sub_*H*_m_) At ⟨*T*⟩, and the Molar Entropies of Sublimation (Δ_sub_*S*_m_) at ⟨*T*⟩ and *p*⟨*T*⟩^b^

		*b*		<*T*>	*p* (<*T*>)	Δ_sub_*H*_m_(<*T*>)	Δ_sub_*S*_m_(<*T*>, *p* (<*T*>))
compound^c^	*a*	K	*r^2^*	K	Pa	kJ·mol^–1^	J·K^–1^·mol^–1^
2-PhI	36.7 ± 0.1	13722 ± 53	0.9997	365.09	0.432	114.1 ± 0.4	312.5 ± 1.2
4-PhI	33.1 ± 0.2	15304 ± 126	0.9991	372.46	0.379	127.2 ± 1.0	341.6 ± 2.8
4,5-DPhI (exp. #1)	38.9 ± 0.1	16900 ± 53	0.99995	421.41	0.304	140.5 ± 0.4	333.4 ± 1.1
4,5-DPhI (exp. #2)	39.3 ± 0.1	17054 ± 30	0.99998	421.40	0.301	141.8 ± 0.2	336.5 ± 0.6
4,5-DPhI (exp. #3)	39.3 ± 0.1	17038 ± 43	0.99997	421.89	0.327	141.7 ± 0.4	335.8 ± 0.8
2,4,5-TPhI (exp. #1)	41.3 ± 0.1	19435 ± 35	0.99999	456.30	0.280	161.6 ± 0.3	354.1 ± 0.6
2,4,5-TPhI (exp. #2)	41.5 ± 0.1	19560 ± 51	0.99997	458.77	0.335	162.6 ± 0.4	354.5 ± 0.9
2,4,5-TPhI (exp. #3)	41.1 ± 0.2	19347 ± 77	0.99995	455.05	0.243	160.9 ± 0.6	353.5 ± 1.4

a The standard uncertainty of the temperature is *u*(*T*/K) = 0.02, and that of the equilibrium vapor pressure is *u*(*p*/Pa)= 0.005. The uncertainties quoted for *a* and *b* are the standard deviations of the fitting parameters.

b The uncertainties quoted are the combined standard uncertainties.

c Vapor pressure measurements for 2-PhI and 4-PhI were conducted using the gravimetric Knudsen effusion methodology. Vapor pressure measurements for 4,5-DPhI and 2,4,5-TPhI, involving three independent experiments, were performed using the combined Knudsen/quartz crystal effusion methodology.

**Table 4:** Standard (*p°* = 10^5^ Pa) Molar Enthalpies (Δ_sub_*H*_m_^°^ and Δ_vap_*H*_m_^°^), Entropies (Δ_sub_*S*_m_^°^ and Δ_vap_*S*_m_^°^), and Gibbs Energies (Δ_sub_*G*_m_^°^ and Δ_vap_*G*_m_^°^) of Sublimation and Vaporization, at *θ* = 298.15 K, for Imidazole (IM), 2-Phenylimidazole (2-PhI), 4-Phenylimidazole (4-PhI), 4,5-Diphenylimidazole (4,5-DPhI), and 2,4,5-Triphenylimidazole (2,4,5-TPhI)^a^

PAHs	ref	Δ_sub_*H*_m_^°^(*θ*) / kJ·mol^–1^	Δ_sub_*S*_m_^°^(*θ*) / J·K^–1^·mol^–1^	Δ_sub_*G*_m_^°^(*θ*) /kJ·mol^–1^	Δ_vap_*H*_m_^°^(*θ*) /kJ·mol^–1^	Δ_vap_*S*_m_^°^(θ) / J·K^–1^·mol^–1^	Δ_vap_*G*_m_^°^(θ) /kJ·mol^–1^
Values at θ = 298.15 K							
IM	ref (59)	82.5 ± 0.1	170.9 ± 0.4	31.5 ± 0.1	71.3 ± 0.4	140.7 ± 1.3	29.4 ± 0.1
2-PhI	this work	115.4 ± 0.6	213.7 ± 1.6	51.7 ± 0.7	104.0 ± 2.6	189.5 ± 7.1	47.5 ± 3.3
4-PhI	this work	128.5 ± 1.1	241.6 ± 3.0	56.5 ± 1.4	114.2 ± 2.5	209.8 ± 7.2	51.7 ± 3.3
4,5-DPhI	this work	143.9 ± 0.8	237.1 ± 2.1	73.2 ± 1.0	119.8 ± 4.3	196.1 ± 10.8	61.4 ± 5.3
2,4,5-TPhI	this work	165.3 ± 1.1	257.6 ± 2.8	88.5 ± 1.4	133.0 ± 5.1	207.2 ± 12.5	71.2 ± 6.4

a The thermodynamic properties of sublimation at *θ* = 298.15 K for 2-PhI, 4-PhI, 4,5-DPhI, and 2,4,5-TPhI were determined from experimental data obtained at ⟨*T*⟩ and *p*⟨*T*⟩ by adjusting for heat capacity differences between the gas and solid phases. The thermodynamic properties of vaporization at *θ* = 298.15 K were determined by combining the thermodynamic properties of sublimation with those of fusion. The uncertainties quoted are the combined standard uncertainties.

For a more comprehensive assessment of the solid phase’s relative stability concerning the sublimation process, it is crucial to analyze thermodynamic properties at the same temperature, in this case, *θ* = 298.15 K. As expected, the incorporation of additional phenyl groups into the imidazole ring led to a reduction in compound volatility. A discernible difference in volatility emerged among the PhI isomers, with 4-PhI exhibiting lower volatility (higher Δ_sub_*G*_m_^°^(*θ*)) compared to its congener, 2-PhI. This distinction arises from a combination of enthalpic and entropic factors. Specifically, the Δ_sub_*H*_m_^°^(*θ*) value of 4-PhI was found to be 13.1 kJ·mol^–1^ greater than the value determined for 2-PhI. An enthalpic–entropic compensation was observed, with a markedly higher value of Δ_sub_*S*_m_^°^(*θ*) for 4-PhI. The experimental data clearly indicate that 4-PhI exhibits stronger intermolecular forces in its crystalline structure, as evidenced by the higher Δ_sub_*H*_m_^°^, coupled with a reduction in the entropy of the crystal, as reflected in the greater Δ_sub_*S*_m_^°^. The introduction of additional phenyl groups led to a clear increase in the magnitude of Δ_sub_*G*_m_^°^(*θ*), where the enthalpic contribution emerged as the predominant factor. The addition of a phenyl group in 4,5-DPhI (resulting in 2,4,5-TPhI) led to an increase in Δ_sub_*H*_m_^°^(*θ*) of 21.4 kJ·mol^–1^. This value is approximately half the contribution of the phenyl group to the increase in Δ_sub_*H*_m_^°^(*θ*) when comparing the compounds biphenyl (Δ_sub_*H*_m_^°^(*θ*) = 81.5 kJ·mol^–1^) and *p*-terphenyl (Δ_sub_*H*_m_^°^(*θ*) = 125.6 kJ·mol^–1^).^72,73^ This differentiation arises from the distinct spatial arrangements of the phenyl groups within the molecular structures. The enthalpic factors contributing to variation in terms of intermolecular interactions can be further examined by deducing the cohesive energies that coexist in both solid and liquid phases. In a liquid displaying a substantial level of structuration, the Δ_fus_*H*_m_^°^(*θ*)/Δ_sub_*H*_m_^°^(*θ*) ratio is generally low, indicating that the majority of intermolecular interactions persist after the melting process.^74^ According to the results obtained, the ratio Δ_fus_*H*_m_^°^(*θ*)/Δ_sub_*H*_m_^°^(*θ*) was found to be 0.11, 0.10, 0,11, 0.17, and 0.20 for IM, 2-PhI, 4-PhI, 4,5-DPhI, and 2,4,5-TPhI, respectively. The IM and the PhI isomers exhibit notably lower and similar ratios, indicating a higher preservation of intermolecular interactions in the liquid state. Conversely, an increase in this ratio is observed with the introduction of extra phenyl groups to the central imidazole ring.

### 3.4 Molecular Structure–Property Correlations

A comparative analysis of the molecular structure, as well as the crystalline packing, of each phenylimidazole is of key relevance, particularly in the context of understanding the thermodynamic differentiation observed in phase equilibrium studies. For the gas phase, the optimized geometries of the studied compounds are presented in the Supporting Information. The insertion of phenyl groups into the imidazole ring was found to introduce steric effects, influencing the overall geometry of the molecule. This was especially evident for 4,5-DPhI and 2,4,5-TPhI. When benzene and imidazole bond to form a phenylimidazole, the interactions between the *ortho* hydrogen atom on the benzene ring with the H–N hydrogen in the imidazole ring (CH···HN) or with the H–C hydrogen in the 5-position of the imidazole ring (CH···HC) are identified as potential contributions to the destabilization of the coplanar structure. Contrary to this expectation, the results reveal a planar structure for 4-PhI and a quasi-planar structure for 2-PhI. This finding has been previously documented by other authors who have explained the coplanar geometry of 4-PhI through fluorescence spectroscopy studies.^75,76^ The fact that 4-PhI exhibits greater planarity in comparison to 2-PhI can be related to the greater CH···HN distance in 4-PhI (2.38 Å) in contrast to the CH···HC distance in 2-PhI (2.21 Å), according to our computational studies. For instance, it is reported that 5-phenylimidazole (the tautomer of 4-PhI) assumes a nonplanar conformation due to an additional repulsive NH···HC interaction.^75^

**Figure 4:**
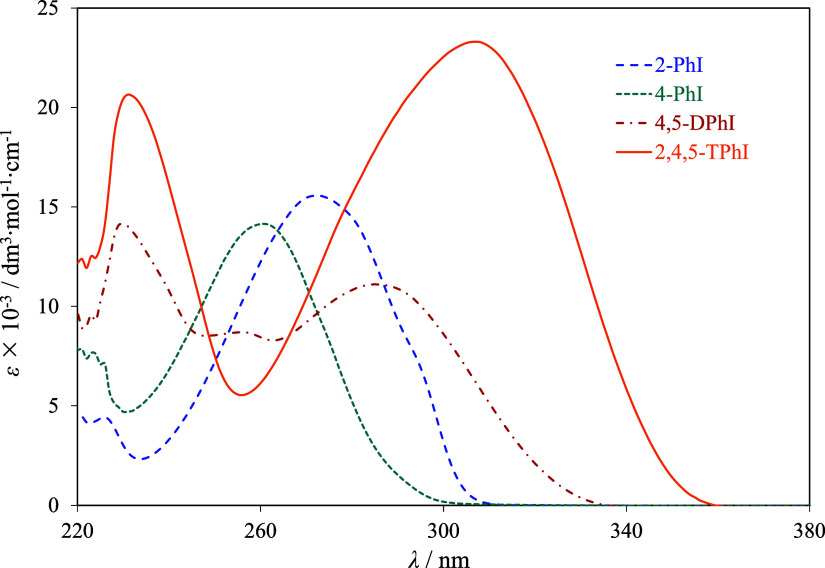
UV–Vis absorption spectra, recorded in CH_2_Cl_2_, at *T* = 298.15 K, for 2-PhI (blue dashed line), 4-PhI (green dashed line), 4,5-DPhI (red dashed line), and 2,4,5-TPhI (orange solid line).

To complement the molecular analysis, Figure 4 presents the experimental UV–vis absorption spectra of the compounds studied. A comparison of the experimental and theoretical UV–vis spectra and the calculated oscillator strengths, the positions of relevant transitions, and frontier orbitals is presented in the SI. The theoretical spectra reasonably agree with the experimental results, although for all compounds, the experimental spectra are red-shifted compared to the theoretical spectra.

According to Figure 4, the insertion of additional phenyl groups into the central imidazole ring resulted in bathochromic shifts in the UV–vis spectrum. The presence of an extra phenyl ring was identified as contributing to extended conjugation. The spectrum of 2-PhI was observed to be red-shifted compared to that of 4-PhI. The HOMO orbital of 4-PhI was reported to extensively delocalize onto the imidazole ring.^75^ While spectroscopic analysis of this nature has not been reported for 2-PhI, it is anticipated that the isomers exhibit varying levels of electronic delocalization in the imidazole ring. For the solid phase, a comprehensive analysis of the supramolecular structures is imperative to interpret the magnitude of the thermodynamic properties of sublimation. These properties offer valuable insights into the strength of intermolecular forces that maintain the substance in its solid state.

**Figure 5:**
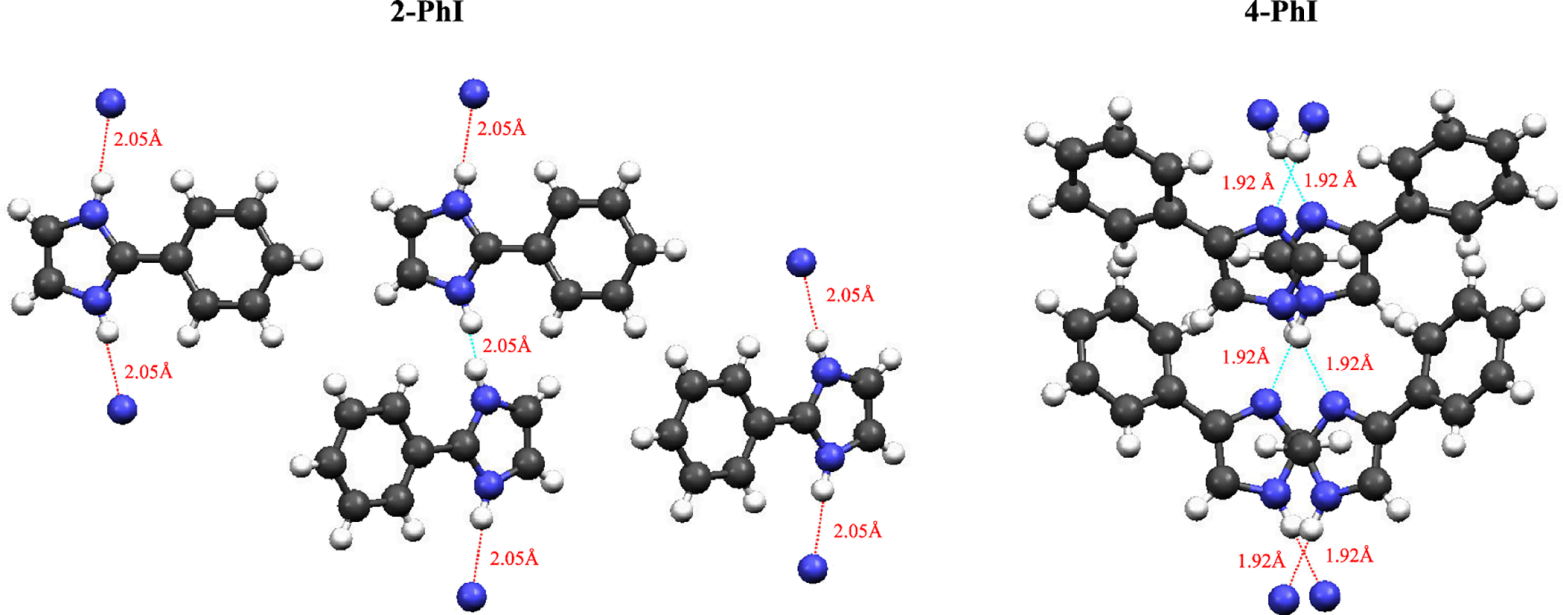
Views of the supramolecular structure of 2-phenylimidazole (2-PhI) and 4-phenylimidazole (4-PhI), highlighting the intermolecular hydrogen bonding interactions.^34,35^

The analysis of the crystalline structures of 2-PhI, 4-PhI, 4,5-DPhI, and 2,4,5-TPhI provides important information on the species’ geometry and offers evidence of the presence of hydrogen bonding.^34−37^ Detailed images of the crystalline packing of each compound are provided in the Supporting Information. Focusing on the PhI isomers, Figure 5 presents views on the supramolecular structures of 2-PhI and 4-PhI. In 2-PhI, the imidazole and phenyl rings are nearly coplanar, and the adjacent molecules are connected through N–H···N hydrogen bonds.^34^ Notably, in contrast to what is observed in the gas phase, the 4-PhI isomer exhibits a clear nonplanar geometry.^35^ The planarity of the molecules is disrupted by strong intermolecular hydrogen bonding, with hydrogen-bond lengths of 1.92 Å observed in 4-PhI, in contrast with a larger value (2.05 Å) found in 2-PhI. The other phenylimidazoles, 4,5-DPhI and 2,4,5-TPhI, were found to establish hydrogen bonding with lengths varying between 2 and 2.05 Å. The smaller hydrogen-bond length observed in 4-PhI points toward a stronger and more direct intermolecular interaction. In comparison to 2-PhI, the supramolecular structure of 4-PhI also displays a more compact arrangement. Furthermore, X-ray data provide evidence that the atoms in 4-PhI exhibit lower molecular motion and disorder compared to 2-PhI.^34,35^ Disordered hydrogen atoms in NH···N hydrogen-bonded chains have been identified in the average crystal structure of 2-PhI.^33^

Table 5 presents some structural properties derived from the crystalline structures reported for 2-PhI, 4-PhI, 4,5,-DPhI, and 2,4,5-TPhI, including the density (ρ) and the molar volume of the unit cell (*V*_m_). Specific (Δ_sub_*h*°) and volumetric (Δ_sub_*h*_v_°) enthalpies of sublimation, expressed as {Δ_sub_*h*° = Δ_sub_*H*° /*M*} and {Δ_sub_*h*_v_° = Δ_sub_*H*° · *ρ*/*M_M_*}, respectively, are also provided.

**Table 5:** Values of the Molecular Weight (*M*_*M*_), Density (ρ), and Molar Volume of the Unit Cell (*V*_m_) for 2-Phenylimidazole (2-PhI), 4-Phenylimidazole (4-PhI), 4,5-Diphenylimidazole (4,5-DPhI), and 2,4,5-Triphenylimidazole (2,4,5-TPhI),^a^ and Derived Specific (Δ_sub_*h*°) and Volumetric (Δ_sub_*h*_v_°) Enthalpies of Sublimation, at *θ* = 298.15 K^b^

properties	2-PhI	4-PhI	4,5-DPhI	2,4,5-TPhI
*M*_*M*_/g·mol^–1^	144.17	144.17	220.27	296.37
*ρ*/g·cm^–3^	1.260	1.188	1.203	1.239
*V*_m_/cm^3^·mol^–1^	114.38	121.40	183.16	239.11
Δ_sub_*h*°/kJ·g^–1^	0.800 ± 0.004	0.891 ± 0.008	0.653 ± 0.003	0.558 ± 0.004
Δ_sub_*h*_v_°/kJ·cm^–3^	1.009 ± 0.003	1.059 ± 0.007	0.786 ± 0.003	0.691 ± 0.003

a Structural properties derived from the crystal structures of 2-PhI, 4-PhI, 4,5-DPhI, and 2,4,5-TPhI.^34−37^

b Specific and volumetric thermodynamic properties of sublimation were determined based on experimental data. The quoted uncertainties represent the combined standard uncertainties.

**Figure 6:**
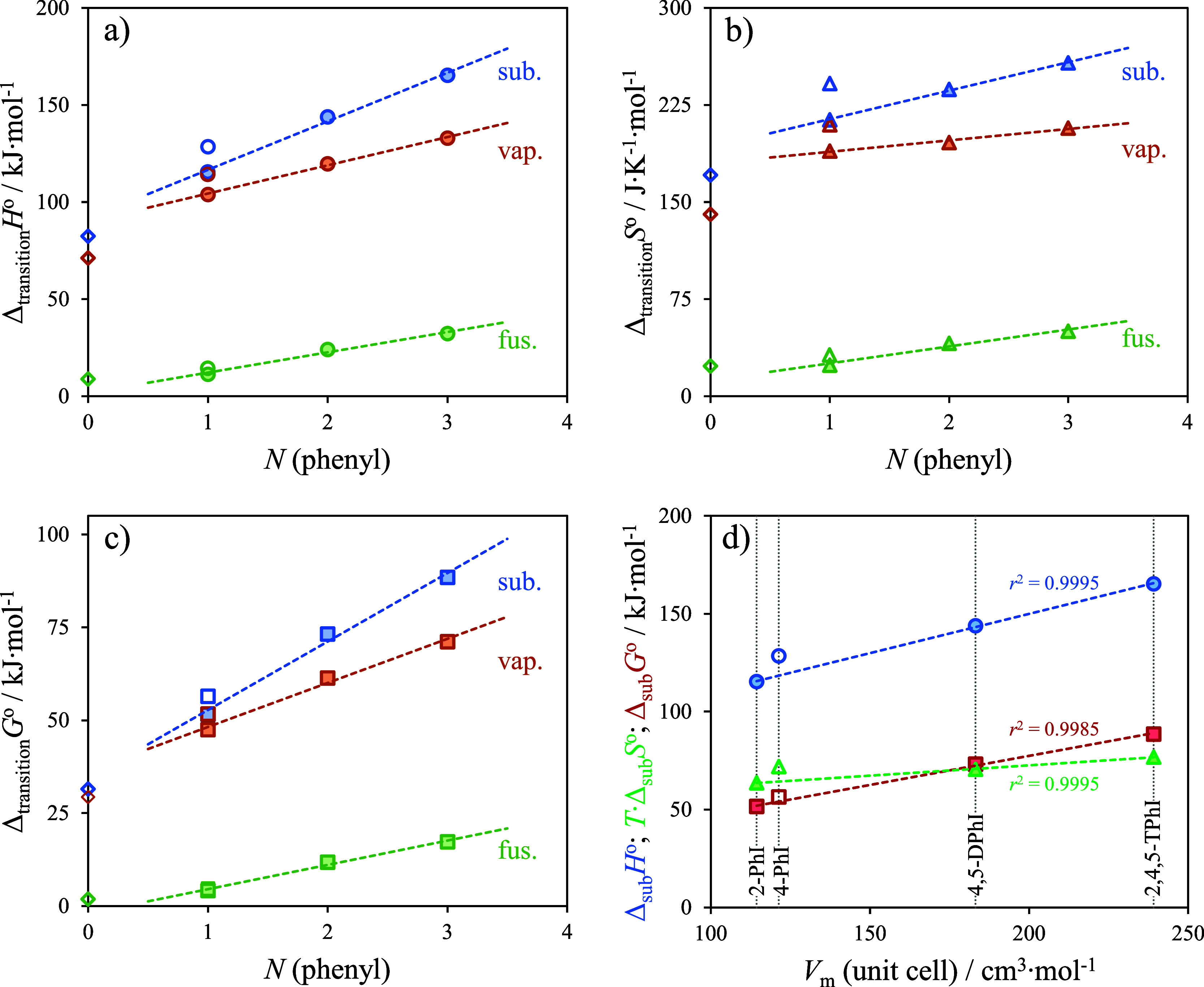
Plots of Δ_transition_*H* = *f* [*N* (phenyl)] (graph a), plots of Δ_transition_*S* = *f* [*N* (phenyl)] (graph b), and plots of Δ_transition_*G* = *f* [*N* (phenyl)] (graph c) for imidazole (IM, *N* = 0), 2-phenylimidazole and 4-phenylimidazole (2-PhI and 4-PhI, *N* = 1), 4,5-diphenylimidazole (4,5-DPhI, *N* = 2), and 2,4,5-triphenylimidazole (2,4,5-TPhI, *N* = 3). Each graph depicts plots of the thermodynamic properties of fusion (fus.), vaporization (vap.), and sublimation (sub.) obtained at the reference temperature of θ = 298.15 K. Graph d presents the plots of the thermodynamic properties of sublimation as a function of the molar volume of the unit cell (*V*_m_), for 2-PhI, 4-PhI, 4,5-DPhI, and 2,4,5-TPhI: Δ_sub_*H* (blue circles); *T*·Δ_sub_*S* (green triangles); Δ_sub_*G* (red squares). The values obtained for IM and 4-PhI (open symbols) are outliers in the linear dependence observed among the values obtained for 2-PhI, 4,5-DPhI, and 2,4,5-TPhI.

4-PhI exhibits both a higher Δ_sub_*h*° and a Δ_sub_*h*_v_° compared to those obtained for its congener, 2-PhI. To enhance the assessment of molecular structure–property correlations, Figure 6 illustrates the plots of thermodynamic properties related to phase transitions (fusion, vaporization, and sublimation) as a function of the number (*N*) of phenyl groups for IM, 2-PhI, 4-PhI, 4,5-DPhI, and 2,4,5-TPhI. The plots of the thermodynamic properties of sublimation as a function of *V*_m_ are also depicted. A primary observation reveals a linear dependence of each property with an increase in the number of phenyl groups. Compared to 4-PhI, the isomer 2-PhI was observed to more closely align with the correlation observed in 4,5-DPhI and 2,4,5-TPhI. This was especially evident in the dependence of Δ_sub_*H*_m_^°^ (graph a) and Δ_sub_*S*_m_^°^ (graph b) with *N*. The values obtained for 4-PhI exceed the predicted values for this compound, considering the fitting of both sublimation and vaporization thermodynamic properties obtained for the other phenylimidazoles. This observation becomes readily apparent when examining the dependence of Δ_sub_*H*_m_^°^, Δ_sub_*S*_m_^°^, Δ_vap_*H*_m_^°^, and Δ_vap_*S*_m_^°^ with *N*.

A less significant deviation is observed in the dependence of Δ_sub_*G*_m_^°^ with *N* (graph c), which can be attributed to the cancellation of effects arising from the enthalpic-entropic compensation. Graph d clearly demonstrates that the thermodynamic properties of sublimation for a homologous series of compounds depend on the magnitude of *V*_m_. A very good correlation (*r*^2^ = 0.9995) was observed in the dependence of Δ_sub_*H*_m_^°^ and *T*·Δ_sub_*S*_m_^°^ on *V*_m_ for 2-PhI, 4,5-DPhI, and 2,4,5-TPhI. Once again, larger values are observed for 4-PhI. In comparison to 2-PhI, the larger Δ_sub_*H*_m_^°^ of 4-PhI arises from the stronger hydrogen bonding, which overall increases the cohesive energies of the lattice. The hydrogen bond network in 4-PhI is probably decreasing its *ρ* relative to the other compounds. The higher structured ordering associated with the hydrogen bond network also helps explaining the larger Δ_sub_*S*_m_^°^ of 4-PhI. Moreover, the greater molecular disorder was observed in the crystal structures of 2-PhI, 4,5-DPhI, and 2,4,5-TPhI.^33,34,36,37^

In summary, the observed trends in heat capacities, coupled with the thermodynamic properties of phase transition, underscore the close relationship between molecular/supramolecular structures and thermodynamic properties. The unique features of 4-PhI, in comparison to 2-PhI, 4,5-DPhI, and 2,4,5-TPhI, arise from distinct supramolecular arrangements facilitated by stronger hydrogen bonding.

The comprehensive thermodynamic characterization presented in this study not only provides new physical insights into the understanding of phenylimidazoles but also establishes a foundation for rationalizing molecular design choices across various applications.

## 4 Conclusions

In conclusion, this investigation systematically explored the structural and thermodynamic properties of a series of phenylimidazoles, namely 2-PhI, 4-PhI, 4,5-DPhI, and 2,4,5-TPhI. The fundamental knowledge of the properties of phenylimidazoles is crucial for various applications extending into the fields of medicinal and materials chemistry. A meticulous analysis of the experimental data has significantly advanced the understanding of how successive phenyl group introductions into the imidazole ring impact the thermodynamic properties and supramolecular characteristics of these compounds.

The insertion of a phenyl group into the imidazole ring resulted in an increment of (85 ± 1) J·K^–1^·mol^–1^ in the *C*_*p*,m_^°^(s) value at *θ* = 298.15 K. According to the phase transition studies, the lower volatility of 4-PhI, in comparison to its isomer 2-PhI, was driven by enthalpy due to an intensified cohesive energy in the solid phase resulting from stronger hydrogen bonding. Very strong N–H···N intermolecular interactions were found to disrupt the coplanarity of the 4-PhI molecules. Additionally, the crystalline structure of 4-PhI, displaying less disorder compared to 2-PhI, and the existence of a hydrogen bond network, explains the larger-than-expected values of the entropies of phase transition (Δ_sub_*S*_m_^°^(*θ*) and Δ_fus_*S*_m_^°^(*θ*)) observed for 4-PhI.

Sublimation data indicated that the addition of a phenyl group in 4,5-DPhI (resulting in 2,4,5-TPhI) led to a Δ_sub_*H*_m_^°^(θ) increase of 21.4 kJ·mol^–1^, about half of the value observed for the linear oligophenyls with an equivalent phenyl group increment. Furthermore, the thermodynamic properties of sublimation were found to exhibit a linear correlation with the molar volume of the unit cell.

## Supporting Information Available

Detailed description of the experimental and computational techniques and methodologies, comprehensive results about the determination of heat capacities, melting, and sublimation properties, and detailed characterization of the molecular/supramolecular structures of the compounds studied and the theoretical UV–vis spectra (PDF)
