# Supplementary material for: The Cohesive Interactions in Phenylimidazoles
Source: J Phys Chem A. 2024 May 30;128(23):4674–84. doi: 10.1021/acs.jpca.4c01589 (PMC11182350; doi:10.1021/acs.jpca.4c01589)
Supplement: Supplementary file 1 — jp4c01589_si_001.pdf [file jp4c01589_si_001.pdf]

Supporting Information (SI)

The Cohesive Interactions in Phenylimidazoles

José C. S. Costa, Ana I. M. C. Lobo Ferreira, Carlos F. R. A. C. Lima, and Luís M. N. B. F. Santos*

CIQUP, Institute of Molecular Sciences (IMS), Department of Chemistry and Biochemistry, Faculty of Science, University of Porto, Rua do Campo Alegre s/n, P4169-007 Porto, Portugal

*Corresponding author

jose.costa@fc.up.pt

Index

1. Heat Capacity Measurements S3

2. DSC/Thermodynamic Properties of Fusion S4

3. Knudsen Effusion/Thermodynamic Properties of Sublimation S7

4. Crystal Structure Analysis S10

5. Computational Results S11

6. UV-Vis Spectroscopy (Experimental and Theoretical Data) S15

7. References S25

1. Heat Capacity Measurements

The heat capacities at θ = 298.15 K (solid phase), of all the studied compounds were measured using a high-precision heat capacity drop calorimeter.[1,2] The calorimeter was calibrated with sapphire (α-Al_2O_3, NIST-RM 720), whose $C^{\circ}_{p,\mathrm{m}}$(α-Al_2O_3, 298.15 K) = (79.03 ± 0.08) $J \cdot K^{-1} \cdot mol^{-1}$.[3] Table S1 presents the standard molar heat capacities at 298.15 K for the phenylimidazoles: m_{sample} represents the mass of sample used in each independent experiment and N_{drop} is the number of drop experiments. The reported uncertainty is twice the standard deviation of the mean and includes the calibration uncertainty.

TABLE S1. Standard molar heat capacity values of the studied compounds at θ = 298.15 K. (ε = 6.6683 ± 0.0174 $W \cdot V^{-1}$)

Compound	m_{sample}/g	N_{drop}	T_{furnace}/K	$T_{\mathrm{calorimeter}}$/K	$<T>$ /K	$C^{\circ}_{p,\mathrm{m}}$/ $J \cdot K^{-1} \cdot mol^{-1}$	$\langle C^{\circ}_{p,\mathrm{m}} \rangle$/ $J \cdot K^{-1} \cdot mol^{-1}$
2-PhI	0.35722	13	303.23	293.18	298.21	169.9 ± 1.5	169.6 ± 1.5
	0.39021	11	303.25	293.18	298.22	169.2 ± 1.5	
4-PhI	0.25420	12	303.25	293.18	298.22	166.2 ± 1.7	167.3 ± 1.3
	0.28860	41	303.24	293.18	298.21	167.6 ± 0.9	
4,5-DPhI	0.22415	38	303.32	293.18	298.25	254.6 ± 2.1	254.3 ± 2.2
	0.20642	20	303.28	293.18	298.23	253.8 ± 2.3	
2,4,5-TPhI	0.15703	23	303.30	293.18	298.24	340.2 ± 3.7	339.8 ± 3.6
	0.20539	10	303.33	293.18	298.26	339.0 ± 3.3	

N_{drop} = number of drop experiments; T_{furnace} = average temperature of the furnace; $T_{\mathrm{calorimeter}}$ = average temperature of the calorimeter; ε = calibration constant; the uncertainty reported is twice the standard deviation of the mean and the calibration uncertainty is included.

2. DSC/Thermodynamic Properties of Fusion

The temperatures and standard molar enthalpies of fusion for the studied compounds were measured using a power compensation differential scanning calorimeter, specifically the PerkinElmer model Pyris Diamond DSC, employing a heating rate of 5 K·min^{-1} and hermetically sealed aluminum crucibles. A constant flow of nitrogen at 20 mL·min^{-1} was applied. The thermograms obtained for 2-PhI, 4-PhI, 4,5-DPhI, and 2,4,5-TPhI are presented in Figure S1.

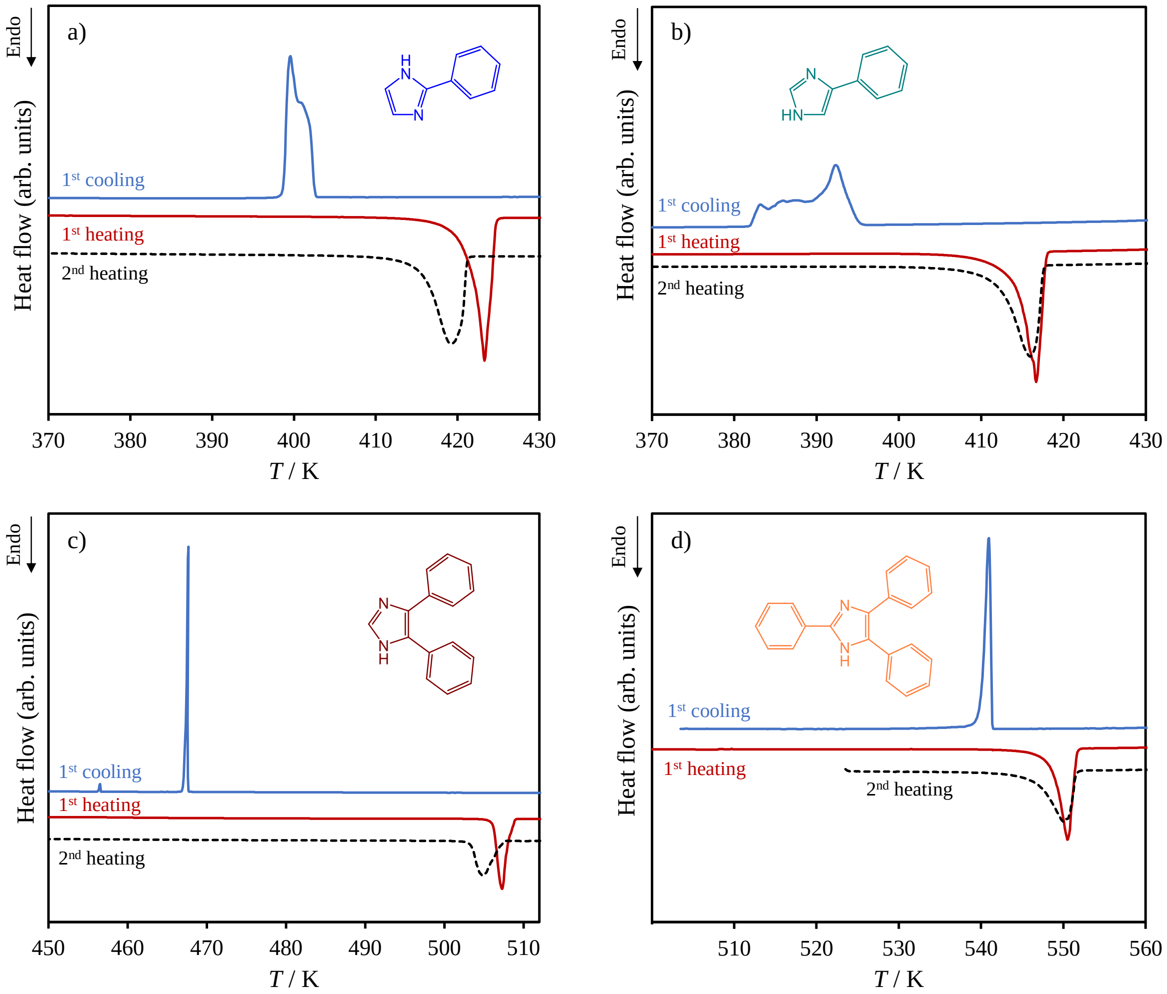

Figure S1. DSC curves for 2-PhI (thermogram a), 4-PhI (thermogram b), 4,5-DPhI (thermogram c), and 2,4,5-TPhI (thermogram d). The results were obtained for samples with a weight range between 5 and 10 mg while applying a scanning rate of 5 K·min^{-1}.

The temperature and heat flux scales were calibrated by measuring the temperature and enthalpy of the fusion of several reference materials: benzoic acid, o-terphenyl, naphthalene, anthracene, 1,3,5-triphenylbenzene, perylene, 1-hexanol, 1-heptanol, diphenylether, and 1,3-difluorobezene.[3-7] The experimental results of 2-PhI, 4-PhI, 4,5-DPhI and 2,4,5-TPhI are presented in Tables S2-S5.

DSC Calibration:

Temperature correction:

$T_{m} = T_{onset} - T_{corr.}$

$T_{corr.} = [(0.00000021809 \times T_{onset}^{3}) + (-0.0002475059 \times T_{onset}^{2}) + (0.09191518 \times T_{onset}) + (-10.10444)]$ K

Energy correction:

$E_{corr.} = A \times k$

$k = 1.0557$

TABLE S2. Detailed results obtained for the study of the fusion equilibrium of 2-PhI (M = 114.17 g·mol^{-1}).

m / mg	T_{onset} / K	A / mJ	T_{m} / K	E / J·g^{-1}	$\Delta_{fus}H^{o}$ / kJ·mol^{-1}	$\Delta_{fus}S^{o}$ / J·K^{-1}·mol^{-1}
6.78	421.3	797	420.3	124.1	17.9	42.6
15.64	422.5	1849	421.5	124.8	18.0	42.7
13.92	422.6	1681	421.6	127.5	18.4	43.6
Final values			**421.1 ± 0.9**		**18.1 ± 1.0**	**43.0 ± 2.4**

The reported uncertainties are twice the standard deviation of the mean and include the calibration uncertainty.

TABLE S3. Detailed results obtained for the study of the fusion equilibrium of 4-PhI (M = 114.17 g·mol^{-1}).

m / mg	T_{onset} / K	A / mJ	T_{m} / K	E / J·g^{-1}	$\Delta_{fus}H^{o}$ / kJ·mol^{-1}	$\Delta_{fus}S^{o}$ / J·K^{-1}·mol^{-1}
4.30	415.3	578	414.3	141.8	20.4	49.3
8.01	415.4	1073	414.5	141.4	20.4	49.2
6.36	415.2	869	414.2	144.2	20.8	50.2
4.55	415.5	617	414.4	143.3	20.7	49.9
9.47	414.9	1285	413.9	143.4	20.7	49.9
Final values			**414.4 ± 0.5**		**20.6 ± 1.0**	**49.7 ± 2.4**

The reported uncertainties are twice the standard deviation of the mean and include the calibration uncertainty.

TABLE S4. Detailed results obtained for the study of the fusion equilibrium of 4,5-DPhI (M = 220.27 g·mol^{-1}).

m / mg	T_{onset} / K	A / mJ	T_m / K	E / J·g^{-1}	$\Delta_{fus}H^o$ / kJ·mol^{-1}	$\Delta_{fus}S^o$ / J·K^{-1}·mol^{-1}
5.46	507.5	834	506.2	161.1	35.5	70.1
7.48	507.4	1148	506.1	162.1	35.7	70.6
6.60	508.9	989	507.5	158.3	34.9	68.7
Final values			**506.6 ± 0.9**		**35.4 ± 1.0**	**69.8 ± 2.0**

The reported uncertainties are twice the standard deviation of the mean and include the calibration uncertainty.

TABLE S5. Detailed results obtained for the study of the fusion equilibrium of 2,4,5-TPhI (M = 296.36 g·mol^{-1}).

m / mg	T_{onset} / K	A / mJ	T_m / K	E / J·g^{-1}	$\Delta_{fus}H^o$ / kJ·mol^{-1}	$\Delta_{fus}S^o$ / J·K^{-1}·mol^{-1}
4.68	550.7	671	548.8	151.6	44.9	81.8
9.26	552.2	1366	550.3	155.8	46.2	83.9
5.53	552.1	812	550.2	155.2	46.0	83.6
9.17	550.3	1375	548.4	158.4	46.9	85.6
Final values			**549.4 ± 0.9**		**46.0 ± 1.0**	**83.7 ± 1.8**

The reported uncertainties are twice the standard deviation of the mean and include the calibration uncertainty.

For each compound, the molar entropy of fusion at T_m was derived according to Equation S1. The molar enthalpies, entropies, and Gibbs energies of fusion at the reference temperature of θ = 298.15 K were calculated according to Equations S2, S3, and S4.

$$\Delta_{fus}S^o = \Delta_{fus}H^o / T_m \qquad \text{(Equation S1)}$$

$$\Delta_{fus}H^o(\theta) = \Delta_{fus}H^o(T_m) + \Delta_{fus}C_p^o \times (\theta - T_m) \qquad \text{(Equation S2)}$$

$$\Delta_{fus}S^o(\theta) = \Delta_{fus}H^o(T_m) / T_m + \Delta_{fus}C_p^o \times \ln(\theta / T_m) \qquad \text{(Equation S3)}$$

$$\Delta_{fus}G^o(\theta) = \Delta_{fus}H^o(\theta) - \theta \times \Delta_{fus}S^o(\theta) \qquad \text{(Equation S4)}$$

In these equations, $\Delta_{fus}C_p^o$ represents the difference between the molar heat capacity of the liquid and the molar heat capacity of the solid at the reference temperature θ. For this analysis, a typical value (recommended by Chickos) of $\Delta_{fus}C_p^o$ = (54.4 ± 20) J·K^{-1}·mol^{-1} was considered.[8] The value of $\Delta_{fus}C_p^o$ was treated as a constant within the temperature range. The temperature θ = 298.15 K was utilized as the reference temperature for this evaluation. The thermodynamic properties at T_m and derived at θ = 298.15 K are presented in Table 2 of the manuscript.

3. Knudsen Effusion/Thermodynamic Properties of Sublimation

For each solid compound, the equilibrium vapor pressures (Table S6), denoted as *p*, were determined at various effusion temperatures using the Knudsen equation (Equation S5).[9,10]

$$p = \frac{\Delta m}{\Delta t \cdot w_o \cdot A_o} \cdot \left(\frac{2 \cdot \pi \cdot R \cdot T}{M}\right)^{1/2} \quad \text{(Equation S5)}$$

$$w_o = \{1 + (3l / 8r)\}^{-1} \quad \text{(Equation S6)}$$

In Equation S5, Δm represents the mass of the sample effused during the experimental time Δt, M stands for the molar mass of the effusing vapor, R denotes the gas constant (8.314462618 $J \cdot mol^{-1} \cdot K^{-1}$), A_o signifies the area of the effusion orifice, and w_o is the transmission probability factor. It was calculated using Equation S6, where l represents the length of the effusion orifice and r its radius.[9,10]

In this technique, only one effusion cell is used, with l = 0.0125 mm and r = 0.600 mm, yielding A_o = 1.1310 mm^2 and w_o = 0.9922. The ln $p = f$ (1 / T) results were fitted using the integrated form of the Clausius-Clapeyron equation (Equation S7).[9,10]

$$\ln(p / p^*) = a - b / T \quad \text{(Equation S7)}$$

where $b = \Delta_{sub}H_m^{\circ}(\langle T \rangle)/R$, and p^* = 1 Pa. Table 3 of the manuscript details the results of fitting the sublimation data to the linear Clausius–Clapeyron equation. The mean temperature, <T>, was determined as the average temperature across all experimental data points; $p(\langle T \rangle)$ represents the pressure at that temperature, obtained from the ln $p = f$ (1/T) linear regression. $\Delta_{sub}S_m^{\circ}(\langle T \rangle, p\langle T \rangle)$ was subsequently calculated according to Equation S8.[9,10]

$$\Delta_{sub}S_m^{\circ}(\langle T \rangle, p\langle T \rangle) = \Delta_{sub}H_m^{\circ}(\langle T \rangle) / \langle T \rangle \quad \text{(Equation S8)}$$

Table S6. Experimental vapor pressure results of crystalline samples (≈ 300 mg) of 2-PhI, 4-PhI, 4,5-DPhI, and 2,4,5-TPhI. Experimental data for 2-PhI and 4-PhI were obtained using the traditional Knudsen effusion technique (gravimetric mass loss detection). Experimental data for 4,5-DPhI and 2,4,5-TPhI were obtained using a combination of a Knudsen effusion cell with a quartz crystal microbalance (QCM).

T/K	*p*/Pa	Δ*p*/Pa	*T*/K	*p*/Pa	Δ*p*/Pa	*T*/K	*p*/Pa	Δ*p*/Pa
				**2-PhI**				
355.25	0.156	0.004	362.14	0.318	0.001	369.02	0.637	-0.007
356.23	0.170	0.001	363.12	0.352	0.001	370.01	0.703	-0.009
357.22	0.191	0.002	364.10	0.386	-0.004	370.99	0.784	-0.001
358.20	0.208	-0.002	365.09	0.431	-0.001	371.97	0.882	0.017
359.18	0.233	0.001	366.07	0.476	-0.002	372.96	0.962	0.008
360.17	0.256	-0.002	367.05	0.525	-0.003	373.94	1.050	-0.001
361.15	0.283	-0.004	368.04	0.579	-0.005	374.92	1.178	0.021
				**4-PhI**				
362.14	0.116	-0.002	369.51	0.264	-0.009	376.89	0.626	0.011
363.61	0.143	0.003	370.99	0.314	-0.008	378.36	0.733	0.013
365.09	0.170	0.005	372.46	0.380	0.001	379.83	0.846	0.004
366.56	0.199	0.003	373.94	0.448	0.002	381.31	0.958	-0.026
368.04	0.228	-0.004	375.41	0.511	-0.013	382.78	1.177	0.029
				**4,5-DPhI**				
	TEST #1			TEST #2			TEST #3	
412.44	0.128	0.001	408.01	0.0804	0.001	409.52	0.0970	0.001
415.44	0.171	0.001	411.02	0.108	0.001	414.47	0.159	0.001
418.44	0.228	-0.001	413.99	0.146	0.001	419.42	0.257	-0.001
421.43	0.304	-0.001	416.97	0.195	-0.001	424.37	0.412	-0.002
424.4	0.402	-0.002	419.93	0.26	-0.001	429.31	0.657	-0.001
427.37	0.533	0.001	422.89	0.346	-0.001	434.26	1.04	0.006
430.35	0.704	0.004	425.85	0.458	-0.001			
			428.82	0.606	0.001			
			431.78	0.798	0.002			
			434.77	1.05	0.005			
				**2,4,5-TPhI**				
	TEST #1			TEST #2			TEST #3	
443.96	0.0856	0.001	446.43	0.103	0.001	445.17	0.0950	0.001
448.87	0.138	0.001	451.36	0.167	0.001	450.11	0.151	-0.001
453.85	0.222	0.001	456.29	0.264	-0.001	455.05	0.243	0.001
458.75	0.353	0.002	461.22	0.418	-0.002	459.99	0.384	0.001
463.7	0.553	0.001	466.18	0.657	-0.002	464.94	0.600	0.001
468.66	0.857	-0.003	471.14	1.03	0.005			

Standard molar properties of sublimation and vaporization at T = 298.15 K

The standard molar enthalpy of sublimation ($\Delta_{\mathrm{sub}}H_{\mathrm{m}}^{o}$, listed in Table 4 of the manuscript) at θ = 298.15 K was determined using Equation S9, considering the negligible dependence of $\Delta_{\mathrm{sub}}H_{\mathrm{m}}$ on pressure and the relation $\Delta_{\mathrm{sub}}C_{p,\mathrm{m}}^{o}$ = $C_{p,m}^{o}$ (g) – $C_{p,m}^{o}$ (s) (values listed in Table 1 of the manuscript).

$$\Delta_{\mathrm{sub}}H_{\mathrm{m}}^{0}\,(\theta) = \Delta_{\mathrm{sub}}H_{\mathrm{m}}\,(\langle T\rangle) + (\theta - \langle T\rangle) \cdot \Delta_{\mathrm{sub}}C_{p,\mathrm{m}}^{0} \qquad \text{(Equation S9)}$$

The standard molar entropy of sublimation, $\Delta_{\mathrm{sub}}S_{\mathrm{m}}^{o}$, at θ = 298.15 K, was calculated according to Equation S10, where $p^{o} = 10^{5}$ Pa.

$$\Delta_{\mathrm{sub}}S_{\mathrm{m}}^{\mathrm{o}}(\theta) = \Delta_{\mathrm{sub}}S_{\mathrm{m}}\big(\langle T\rangle, p(\langle T\rangle)\big) + \Delta_{\mathrm{sub}}C_{p,\mathrm{m}}^{0} \cdot \ln(\theta/\langle T\rangle) - R \cdot \ln\{p^{0}/p(\langle T\rangle)\} \qquad \text{(Equation S10)}$$

The standard molar Gibbs Energy of sublimation, $\Delta_{\mathrm{sub}}G_{\mathrm{m}}^{o}$, at θ = 298.15 K, was calculated according to Equation S11.

$$\Delta_{\mathrm{sub}}G_{\mathrm{m}}^{\mathrm{o}}(\theta) = \Delta_{\mathrm{sub}}H_{\mathrm{m}}^{\mathrm{o}}(\theta) - \theta \times \Delta_{\mathrm{sub}}S_{\mathrm{m}}^{\mathrm{o}}(\theta) \qquad \text{(Equation S11)}$$

The thermodynamic properties of vaporization at θ = 298.15 K (listed in Table 4 of the manuscript) were derived by combining the fusion and sublimation results according to Equations S12, S13, and S14.

$$\Delta_{\mathrm{vap}}H_{\mathrm{m}}^{\mathrm{o}}(\theta) = \Delta_{\mathrm{sub}}H_{\mathrm{m}}^{\mathrm{o}}(\theta) - \Delta_{\mathrm{fus}}H_{\mathrm{m}}^{\mathrm{o}}(\theta) \qquad \text{(Equation S12)}$$

$$\Delta_{\mathrm{vap}}S_{\mathrm{m}}^{\mathrm{o}}(\theta) = \Delta_{\mathrm{sub}}S_{\mathrm{m}}^{\mathrm{o}}(\theta) - \Delta_{\mathrm{fus}}S_{\mathrm{m}}^{\mathrm{o}}(\theta) \qquad \text{(Equation S13)}$$

$$\Delta_{\mathrm{vap}}G_{\mathrm{m}}^{\mathrm{o}}(\theta) = \Delta_{\mathrm{sub}}G_{\mathrm{m}}^{\mathrm{o}}(\theta) - \Delta_{\mathrm{fus}}G_{\mathrm{m}}^{\mathrm{o}}(\theta) \qquad \text{(Equation S14)}$$

4. Crystal Structure Analysis

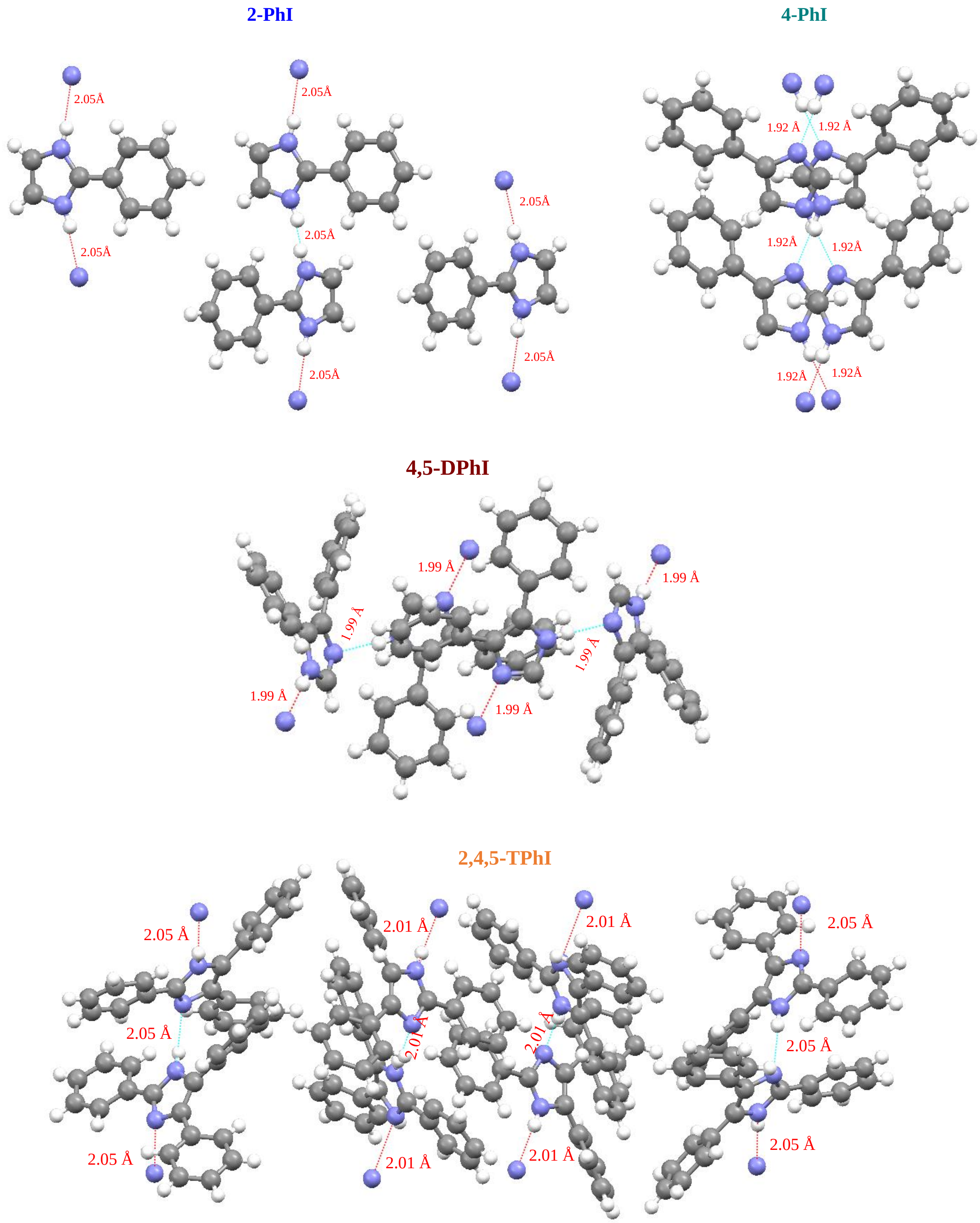

Figure S2. Views of the supramolecular structure of 2-PhI, 4-PhI, 4,5-DPhI, and 2,4,5-TPhI, highlighting the intermolecular hydrogen bonding interactions.[11-14]

5. Computational Results

Full geometry optimizations and frequency calculations, without symmetry restrictions, were conducted at the M06-2X/6-311++G(d,p) level of theory. No imaginary frequencies were found, confirming that the optimized structures correspond to true minima. These calculations were used to determine the gaseous phase heat capacities for all species at 298.15 K and evaluated gas phase molecular geometries and energetics. Comprehensive computational results are provided in Table S7. The optimized geometry of each molecule is presented in Figure S3 and Table S8.

Table S7. Computationally calculated electronic energies at T = 0 K, E_{el}, enthalpies at T = 298.15 K, H_{298}, and molar isobaric heat capacities at T = 298.15 K, $C_{p,\mathrm{m}}$, using the M06-2X/6-311++G(d,p) level of theory, for the molecules studied in vacuum.

Molecule	E_{el} / Hartree	H_{298} / Hartree	$C_{p,\mathrm{m}}$ / J·K^{-1}·mol^{-1}	Imaginary frequencies?
Imidazole	−226.187264	−226.110899	66.46	No
2-Phenylimidazole	−457.208604	−457.046198	150.23	No
4-Phenylimidazole	−457.207529	−457.044991	150.08	No
4,5-Diphenylimidazole	−688.226094	−687.977565	233.20	No
2,4,5-Triphenylimidazole	−919.247788	−918.913158	317.11	No

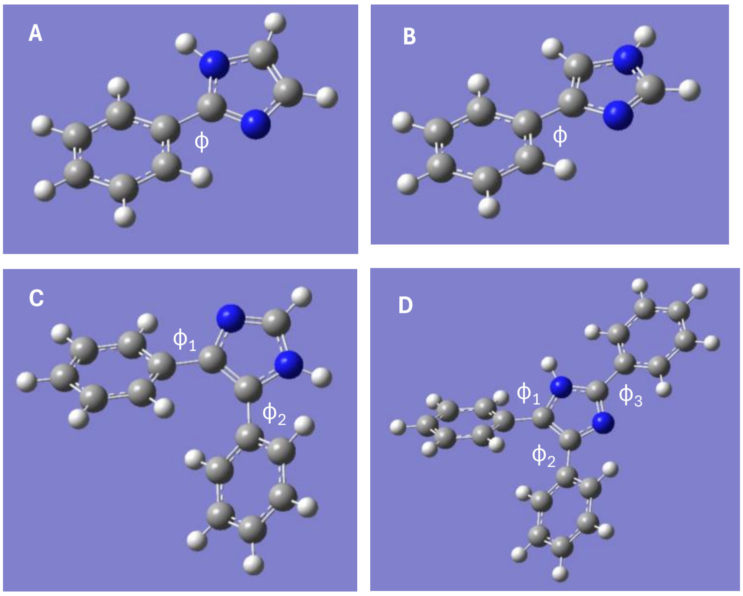

Figure S3. Optimized geometries, at the M06-2X/6-311++G(d,p) level for (in parenthesis are shown the respective dihedral angles): (A) 2-phenylimidazole (φ = 14 º); (B) 4-phenylimidazole (φ = 0 º); (C) 4,5-diphenylimidazole ($φ_1$ = 33 º, $φ_2$ = 42 º); and (D) 2,4,5-triphenylimidazole ($φ_1$ = 41 º, $φ_2$ = 32 º, $φ_3$ = 15 º).

Table S8. Optimized geometries, at the M06-2X/6-311++G(d,p), in Cartesian coordinates.

Imidazole

C	-0.989690	-0.529036	-0.000044
C	1.130583	-0.293509	0.000250
C	0.619719	0.974597	-0.000145
N	-0.745419	0.810768	0.000135
H	-1.435476	1.543587	0.000361
H	1.083516	1.945758	-0.000816
N	0.118478	-1.221903	-0.000214
H	2.166948	-0.590613	0.000517
H	-1.990068	-0.933097	0.000124

2-Phenylimidazole

C	1.030167	-0.057189	-0.016738
C	3.063803	-0.728825	-0.105025
C	3.131265	0.621334	0.104528
N	1.824182	1.040841	0.156076
H	1.511755	1.978610	0.346613
H	3.958464	1.300482	0.218683
N	1.759616	-1.138563	-0.176734
H	3.880535	-1.424647	-0.210224
C	-0.436990	-0.011111	-0.012387
C	-1.140185	1.190841	-0.117000
C	-1.147487	-1.209698	0.094722
C	-2.530149	1.197039	-0.099263
H	-0.613071	2.131605	-0.235461
C	-2.534969	-1.200207	0.107248
H	-0.590566	-2.135815	0.166992
C	-3.231827	0.002128	0.014442
H	-3.064077	2.136067	-0.183879
H	-3.076363	-2.134987	0.192610
H	-4.315035	0.006855	0.026102

4-Phenylimidazole

C	2.993264	-0.778778	-0.000251
C	1.010979	0.036234	0.000043
C	1.866072	1.111607	0.000193
N	3.130192	0.575337	0.000194
H	3.995857	1.089430	-0.000301
H	1.698713	2.174801	0.000369
N	1.738179	-1.134299	-0.000205
H	3.841912	-1.445407	-0.000327
C	-0.459577	0.026123	0.000049
C	-1.143672	-1.191468	0.000196
C	-1.195688	1.214430	-0.000078
C	-2.533113	-1.217438	0.000123
H	-0.568537	-2.109112	0.000395
C	-2.583955	1.186537	-0.000164
H	-0.681997	2.169574	0.000022
C	-3.259285	-0.030755	-0.000096
H	-3.051524	-2.169233	0.000243
H	-3.140687	2.116369	-0.000244
H	-4.342480	-0.052638	-0.000167

4,5-Diphenylimidazole

C	-0.090932	3.301562	-0.032316
C	-0.719460	1.254214	0.026534
C	0.660941	1.222840	-0.000339
N	1.041127	2.548271	-0.018577
H	1.988344	2.880662	-0.110830
N	-1.163353	2.557920	-0.003169
H	-0.068213	4.380217	-0.058878
C	-1.673028	0.134596	0.100387
C	-2.921031	0.254636	-0.516743
C	-1.370677	-1.040615	0.794626
C	-3.837130	-0.788543	-0.460909
H	-3.160009	1.176848	-1.032789
C	-2.287454	-2.083258	0.847130
H	-0.418616	-1.132495	1.305157
C	-3.522162	-1.963147	0.215924
H	-4.800818	-0.683711	-0.945870
H	-2.041630	-2.986695	1.393043
H	-4.237397	-2.775958	0.260500
C	1.648770	0.138405	-0.068301
C	2.831264	0.197851	0.675285
C	1.426886	-0.962037	-0.902650
C	3.773280	-0.821625	0.585484
H	3.001562	1.032335	1.347761
C	2.363450	-1.983024	-0.981744
H	0.514697	-1.006195	-1.486577
C	3.541045	-1.915299	-0.240749
H	4.683556	-0.766118	1.170828
H	2.178807	-2.830096	-1.631740
H	4.272735	-2.711468	-0.308099

2,4,5-Triphenylimidazole

C	-1.698461	0.031507	-0.031447
C	0.345647	0.705403	-0.060495
C	0.405758	-0.674572	-0.011723
N	-0.910379	-1.082630	-0.017179
H	-1.217254	-2.030467	0.137634
N	-0.963734	1.118905	-0.061131
C	1.446855	1.679800	-0.136018
C	1.292521	2.937767	0.452782
C	2.638279	1.387069	-0.806237
C	2.317574	3.873779	0.392911
H	0.358512	3.168043	0.951199
C	3.662791	2.323746	-0.862834
H	2.757036	0.426698	-1.295194
C	3.508199	3.568805	-0.260006
H	2.186278	4.845135	0.855732
H	4.579215	2.085271	-1.389919
H	4.306851	4.299588	-0.308434
C	1.505301	-1.640726	0.092111
C	1.471565	-2.838544	-0.628751
C	2.591341	-1.387968	0.937012
C	2.501304	-3.765238	-0.506528
H	0.648674	-3.031978	-1.309138
C	3.622294	-2.310291	1.048987
H	2.615795	-0.464833	1.504592
C	3.579918	-3.503063	0.330646
H	2.465387	-4.687222	-1.074733
H	4.457418	-2.102659	1.707474
H	4.383765	-4.223374	0.423950
C	-3.164373	-0.010935	-0.014226
C	-3.869383	-1.185723	-0.286020
C	-3.871513	1.157886	0.280606
C	-5.258666	-1.196202	-0.249632
H	-3.341337	-2.095696	-0.550766
C	-5.258599	1.144491	0.311369
H	-3.313737	2.064240	0.481627
C	-5.957323	-0.031910	0.050771
H	-5.795075	-2.112606	-0.464898
H	-5.798644	2.055067	0.542274
H	-7.040270	-0.039618	0.077110

6. UV-Vis Spectroscopy (experimental and theoretical data)

UV–vis spectra (experimental and theoretical) of 2-PhI, 4-PhI, 4,5-DPhI, and 2,4,5-TPhI in CH_2Cl_2 solutions (concentrations of approximately 10^{-5} mol·dm^{-3}) are presented in Figures S4, S5, S6, and S7. Oscillator strength, the position of the transitions, and frontier orbitals are also depicted.

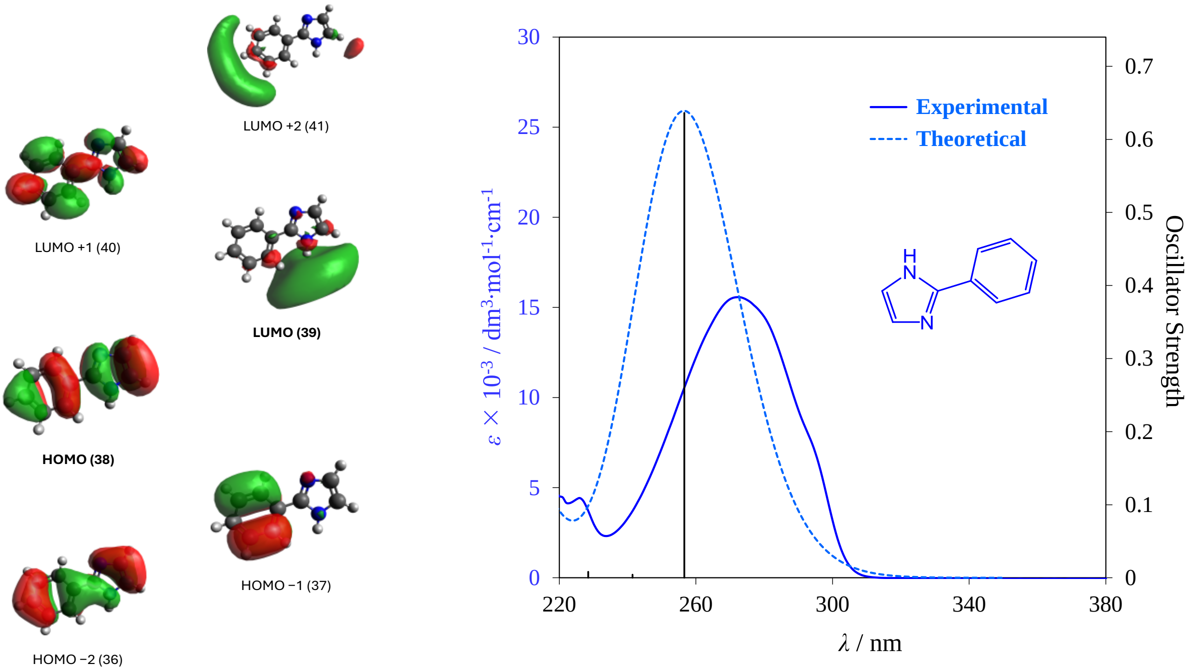

Figure S4. Comparison of the experimental (solid line) and theoretical (dashed line) UV-Vis spectra of 2-PhI, recorded in CH_2Cl_2, at T = 298.1 K. Oscillator strength, the position of relevant transitions, and frontier orbitals are also depicted. TD-DFT electronic absorption spectra were computed at the M06-2X/6-311++G(d,p) level of theory.

Relevant transitions:
HOMO > LUMO (π > σ*);
HOMO > LUMO+1 (π > π*);
HOMO > LUMO+2 (π > σ*);
HOMO-1 > LUMO (π > σ*);
HOMO-1 > LUMO+2 (π > σ*);
HOMO-2 > LUMO (π > σ*);
HOMO-2 > LUMO+1 (π > π*);
HOMO-2 > LUMO+2 (π > σ*).

Table S9. Calculated excitation energies and oscillator strengths, using TD-DFT at the M06-2X/6-311++G(d,p) level of theory, in dichloromethane for 2-PhI.

	Transition	Energy / eV	Wavelength / nm	Oscillator strength
Excited State 1	HOMO > LUMO	4.8318	256.60	f=0.6362
Excited State 2	HOMO-2 > LUMO+3;<br>HOMO-1 > LUMO;<br>HOMO > LUMO+2;<br>HOMO > LUMO+3	5.1354	241.43	f=0.0043
Excited State 3	HOMO > LUMO+1;<br>HOMO > LUMO+2	5.4271	228.46	f=0.0083
Excited State 4	HOMO-2 > LUMO+1;<br>HOMO-1 > LUMO;<br>HOMO > LUMO+1;<br>HOMO > LUMO+2;<br>HOMO > LUMO+3;<br>HOMO > LUMO+5	6.0084	206.35	f=0.0196
Excited State 5	HOMO-1 > LUMO;<br>HOMO > LUMO+3;<br>HOMO > LUMO+4	6.0358	205.41	f=0.1185
Excited State 6	HOMO-3 > LUMO;<br>HOMO-1 > LUMO;<br>HOMO > LUMO+3;<br>HOMO > LUMO+4;<br>HOMO > LUMO+6	6.0866	203.70	f=0.0544
Excited State 7	HOMO-4 > LUMO;<br>HOMO-3 > LUMO;<br>HOMO-3 > LUMO+16;<br>HOMO > LUMO+4	6.2303	199.00	f=0.0068
Excited State 8	HOMO-2 > LUMO;<br>HOMO-1 > LUMO+2;<br>HOMO-1 > LUMO+3;<br>HOMO > LUMO+5;<br>HOMO > LUMO+6;<br>HOMO > LUMO+7	6.3893	194.05	f=0.2045

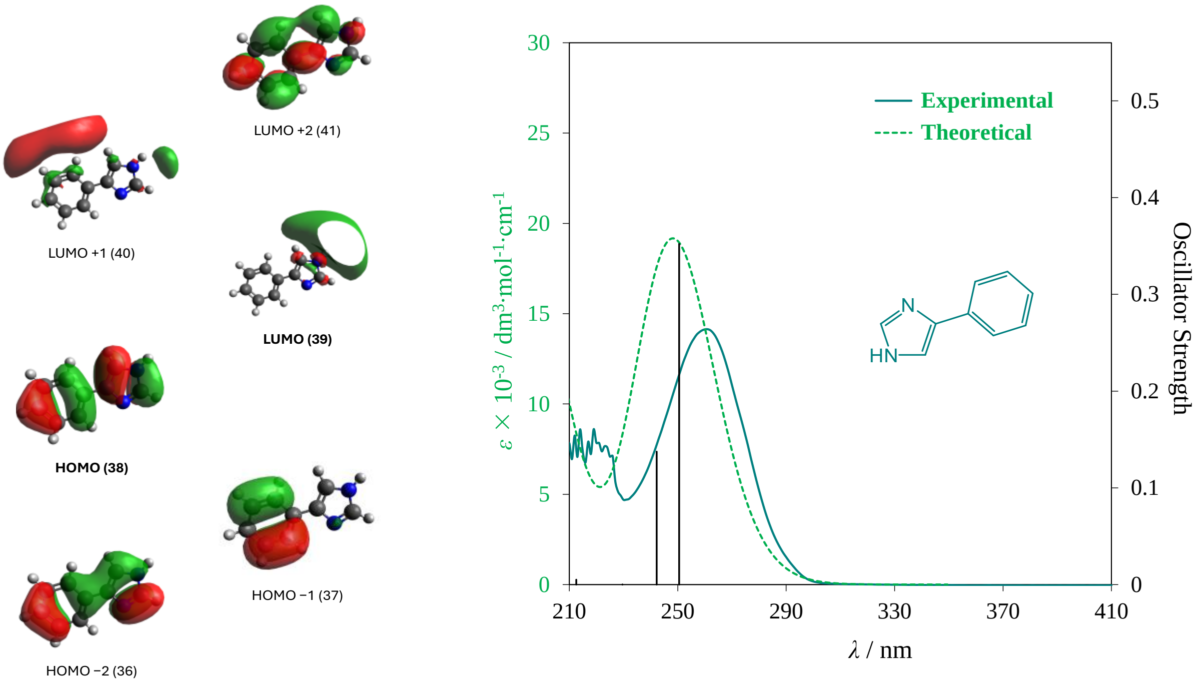

Figure S5. Comparison of the experimental (solid line) and theoretical (dashed line) UV-Vis spectra of 4-PhI, recorded in CH_2Cl_2, at T = 298.1 K. Oscillator strength, the position of relevant transitions, and frontier orbitals are also depicted. TD-DFT electronic absorption spectra were computed at the M06-2X/6-311++G(d,p) level of theory.

Relevant transitions:
HOMO > LUMO (π > σ*);
HOMO > LUMO+1 (π > σ*);
HOMO > LUMO+2 (π > π*);
HOMO-1 > LUMO (π > σ*);
HOMO-2 > LUMO+1 (π > σ*);
HOMO-2 > LUMO+2 (π > π*).

Table S10. Calculated excitation energies and oscillator strengths, using TD-DFT at the M06-2X/6-311++G(d,p) level of theory, in dichloromethane for 4-PhI.

	Transition	Energy / eV	Wavelength / nm	Oscillator strength
Excited State 1	HOMO-1 > LUMO;<br>HOMO > LUMO;<br>HOMO > LUMO+4	4.9497	250.49	f=0.3522
Excited State 2	HOMO-2 > LUMO+4;<br>HOMO-1 > LUMO;<br>HOMO > LUMO;<br>HOMO > LUMO+4	5.1184	242.23	f=0.1375
Excited State 3	HOMO > LUMO+1;<br>HOMO > LUMO+9	5.4004	229.58	f=0.0002
Excited State 4	HOMO-2 > LUMO+1;<br>HOMO > LUMO+2;<br>HOMO > LUMO+5	5.8335	212.54	f=0.0050
Excited State 5	HOMO-2 > LUMO+4;<br>HOMO-1 > LUMO;<br>HOMO > LUMO+4;<br>HOMO > LUMO+7;<br>HOMO > LUMO+11;<br>HOMO > LUMO+13	5.9476	208.46	f=0.1210
Excited State 6	HOMO > LUMO+3	6.0267	205.73	f=0.0027
Excited State 7	HOMO-1 > LUMO;<br>HOMO > LUMO+4;<br>HOMO > LUMO+7	6.3001	196.80	f=0.3105
Excited State 8	HOMO-2 > LUMO+2;<br>HOMO > LUMO+1;<br>HOMO > LUMO+2;<br>HOMO > LUMO+5;<br>HOMO > LUMO+6;<br>HOMO > LUMO+9;<br>HOMO > LUMO+10	6.3265	195.97	f=0.0022

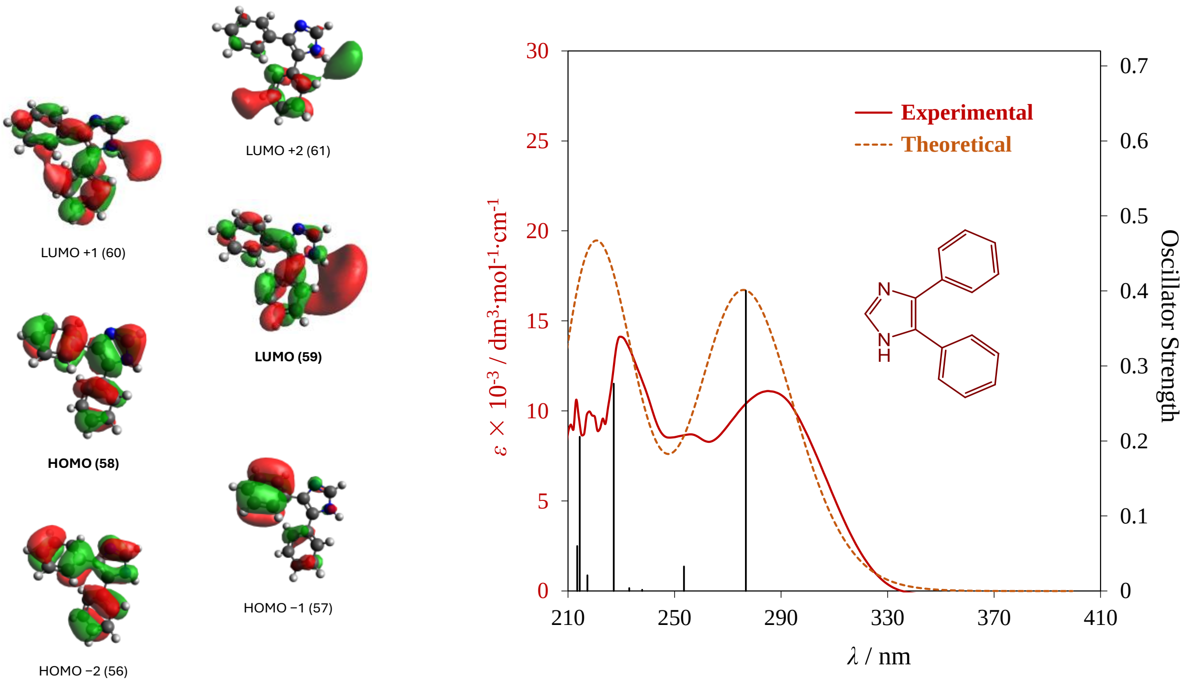

Figure S6. Comparison of the experimental (solid line) and theoretical (dashed line) UV-Vis spectra of 4,5-DPhI, recorded in CH_2Cl_2, at T = 298.1 K. Oscillator strength, the position of relevant transitions, and frontier orbitals are also depicted. TD-DFT electronic absorption spectra were computed at the M06-2X/6-311++G(d,p) level of theory.

Relevant transitions:
HOMO > LUMO (π > π*);
HOMO > LUMO+1 (π > π*);
HOMO > LUMO+2 (π > π*);
HOMO-1 > LUMO (π > π*);
HOMO-1 > LUMO+2 (π > π*);
HOMO-2 > LUMO (π > π*);
HOMO-2 > LUMO+2 (π > π*).

Table S11. Calculated excitation energies and oscillator strengths, using TD-DFT at the M06-2X/6-311++G(d,p) level of theory, in dichloromethane for 4,5-DPhI.

	Transition	Energy / eV	Wavelength / nm	Oscillator strength
Excited State 1	HOMO > LUMO	4.4796	276.78	f=0.4002
Excited State 2	HOMO-2 > LUMO;<br>HOMO-2 > LUMO+8;<br>HOMO-1 > LUMO;<br>HOMO > LUMO+1;<br>HOMO > LUMO+2;<br>HOMO > LUMO+3	4.8902	253.53	f=0.0328
Excited State 3	HOMO-3 > LUMO;<br>HOMO-2 > LUMO+2;<br>HOMO-1 > LUMO;<br>HOMO-1 > LUMO+2;<br>HOMO-1 > LUMO+5;<br>HOMO-1 > LUMO+6;<br>HOMO > LUMO+2;<br>HOMO > LUMO+5;<br>HOMO > LUMO+6;<br>HOMO > LUMO+7;<br>HOMO > LUMO+8	5.2141	237.79	f=0.0017
Excited State 4	HOMO-1 > LUMO;<br>HOMO > LUMO+1;<br>HOMO > LUMO+2;<br>HOMO > LUMO+4;<br>HOMO > LUMO+10	5.3218	232.97	f=0.0038
Excited State 5	HOMO > LUMO+5;<br>HOMO > LUMO+6;<br>HOMO > LUMO+8	5.4582	227.15	f=0.2764
Excited State 6	HOMO-1 > LUMO;<br>HOMO > LUMO+2;<br>HOMO > LUMO+3;<br>HOMO > LUMO+5;<br>HOMO > LUMO+10	5.7073	217.24	f=0.0208
Excited State 7	HOMO-4 > LUMO+2;<br>HOMO-3 > LUMO;<br>HOMO-3 > LUMO+5,<br>HOMO-3 > LUMO+6;<br>HOMO-2 > LUMO;<br>HOMO-1 > LUMO;<br>HOMO > LUMO+2;<br>HOMO > LUMO+3<br>HOMO > LUMO+4;<br>HOMO > LUMO+5;<br>HOMO > LUMO+6	5.7828	214.40	f=0.2056
Excited State 8	HOMO-1 > LUMO;<br>HOMO > LUMO+1;<br>HOMO > LUMO+3;<br>HOMO > LUMO+4;<br>HOMO > LUMO+5;<br>HOMO > LUMO+6;<br>HOMO > LUMO+11;<br>HOMO > LUMO+15	5.8084	213.45	f=0.0599

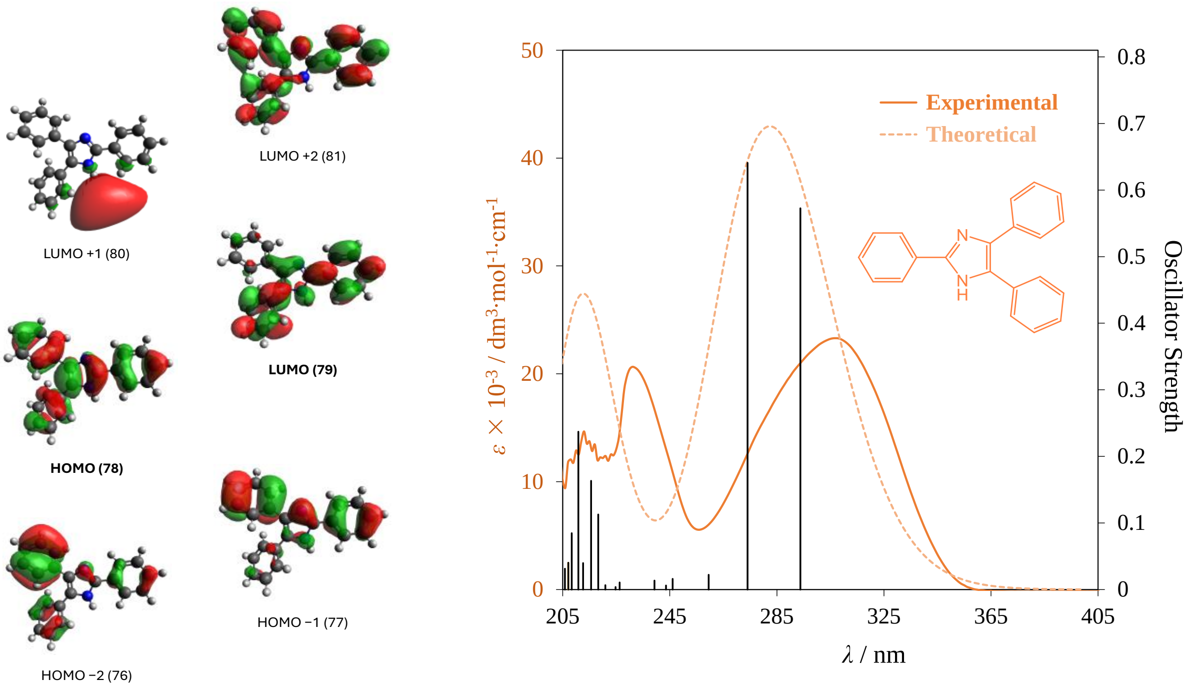

Figure S7. Comparison of the experimental (solid line) and theoretical (dashed line) UV-Vis spectra of 2,4,5-TPhI, recorded in CH_2Cl_2, at T = 298.1 K. Oscillator strength, the position of relevant transitions, and frontier orbitals are also depicted. TD-DFT electronic absorption spectra were computed at the M06-2X/6-311++G(d,p) level of theory.

Relevant transitions:
HOMO > LUMO (π > π*);
HOMO > LUMO+1 (π > σ*);
HOMO > LUMO+2 (π > π*);
HOMO-1 > LUMO (π > π*);
HOMO-1 > LUMO+1 (π > σ*);
HOMO-2 > LUMO (π > π*);
HOMO-2 > LUMO+1 (π > σ*).

Table S12. Calculated excitation energies and oscillator strengths, using TD-DFT at the M06-2X/6-311++G(d,p) level of theory, in dichloromethane for 2,4,5-TPhI.

	Transition	Energy / eV	Wavelength / nm	Oscillator strength
Excited State 1	HOMO > LUMO;<br>HOMO > LUMO+1	4.2199	293.81	f=0.5726
Excited State 2	HOMO > LUMO;<br>HOMO > LUMO+1	4.5243	274.04	f=0.6407
Excited State 3	HOMO-2 > LUMO+1;<br>HOMO > LUMO+2;<br>HOMO > LUMO+3;<br>HOMO > LUMO+4;<br>HOMO > LUMO+5	4.7772	259.53	f=0.0222
Excited State 4	HOMO-4 > HOMO-3;<br>HOMO-3 > LUMO;<br>HOMO-3 > LUMO+1;<br>HOMO-1 > LUMO+6;<br>HOMO > LUMO+2;<br>HOMO > LUMO+6	5.0391	2LUMO+7.04	f=0.0160
Excited State 5	HOMO-3 > LUMO;<br>HOMO > LUMO+2;<br>HOMO > LUMO+3;<br>HOMO > LUMO+4;<br>HOMO > LUMO+5;<br>HOMO > LUMO+6;<br>HOMO > LUMO+8;<br>HOMO > LUMO+10	5.0897	243.60	f=0.0059
Excited State 6	HOMO-5 > LUMO;<br>HOMO-5 > LUMO+1;<br>HOMO-2 > LUMO+1;<br>HOMO-2 > LUMO+12;<br>HOMO-1 > LUMO+10;<br>HOMO-1 > LUMO+11;<br>HOMO > LUMO+2;<br>HOMO > LUMO+10;<br>HOMO > LUMO+11	5.1821	239.25	f=0.0135
Excited State 7	HOMO > LUMO+3;<br>HOMO > LUMO+7;<br>HOMO > LUMO+8;<br>HOMO > LUMO+9;<br>HOMO > LUMO+10;<br>HOMO > LUMO+11;<br>HOMO > LUMO+12	5.4793	226.28	f=0.0107
Excited State 8	HOMO > LUMO+3;<br>HOMO > LUMO+5;<br>HOMO > LUMO+7;<br>HOMO > LUMO+8;<br>HOMO > LUMO+15	5.5166	224.75	f=0.0037
Excited State 9	HOMO > LUMO+3;<br>HOMO > LUMO+4;<br>HOMO > LUMO+5;<br>HOMO > LUMO+8;<br>HOMO > LUMO+12	5.6122	220.92	f=0.0064

Excited State	10	HOMO-5 > LUMO;<br>HOMO-5 > LUMO+12;<br>HOMO-4 > LUMO;<br>HOMO-3 > LUMO;<br>HOMO-2 > LUMO;<br>HOMO-2 > LUMO+1;<br>HOMO > LUMO+4;<br>HOMO > LUMO+5;<br>HOMO > LUMO+12	5.6804	218.27	f=0.1126
Excited State	11	HOMO-3 > LUMO;<br>HOMO-3 > LUMO+1;<br>HOMO-1 > LUMO;<br>HOMO > LUMO+5;<br>HOMO > LUMO+6;<br>HOMO > LUMO+7;<br>HOMO > LUMO+8;<br>HOMO > LUMO+9;<br>HOMO > LUMO+11;<br>HOMO > LUMO+12;<br>HOMO > LUMO+13	5.7517	215.56	f=0.1632
Excited State	12	HOMO-3 > LUMO;<br>HOMO-3 > LUMO+1;<br>HOMO-1 > LUMO;<br>HOMO > LUMO+5;<br>HOMO > LUMO+6;<br>HOMO > LUMO+8;<br>HOMO > LUMO+10;<br>HOMO > LUMO+12;<br>HOMO > LUMO+13	5.8322	212.59	f=0.0396
Excited State	13	HOMO-4 > LUMO+1;<br>HOMO-3 > LUMO;<br>HOMO-3 > LUMO+1;<br>HOMO-2 > LUMO+1;<br>HOMO-1 > LUMO;<br>HOMO-1 > LUMO+1;<br>HOMO > LUMO+6;<br>HOMO > LUMO+7;<br>HOMO > LUMO+11	5.8801	210.85	f=0.2371
Excited State	14	HOMO-5 > LUMO;<br>HOMO-5 > LUMO+1;<br>HOMO-4 > LUMO;<br>HOMO-2 > LUMO+1;<br>HOMO-1 > LUMO;<br>HOMO > LUMO+2;<br>HOMO > LUMO+7;<br>HOMO > LUMO+8;<br>HOMO > LUMO+10;<br>HOMO > LUMO+11	5.9502	208.37	f=0.0845
Excited State	15	HOMO-5 > LUMO;<br>HOMO-5 > LUMO+1;<br>HOMO-4 > LUMO;<br>HOMO-2 > LUMO;<br>HOMO-2 > LUMO+1;<br>HOMO > LUMO+2;	5.9879	207.06	f=0.0402

	HOMO > LUMO+7;<br>HOMO > LUMO+8;<br>HOMO > LUMO+9;<br>HOMO > LUMO+10;<br>HOMO > LUMO+11;<br>HOMO > LUMO+16			
Excited State 16	HOMO-7 > LUMO;<br>HOMO-7 > LUMO+1;<br>HOMO-6 > LUMO;<br>HOMO-5 > LUMO;<br>HOMO-4 > LUMO;<br>HOMO-3 > LUMO+6;<br>HOMO-1 > LUMO+1;<br>HOMO > LUMO+7;<br>HOMO > LUMO+9	6.0243	205.81	f=0.0313

References

1. Santos, L. M. N. B. F.; Rocha, M. A. A.; Rodrigues, A. S. M. C.; Štejfa, V.; Fulem, M.; Bastos, M. Reassembling and Testing of a High-Precision Heat Capacity Drop Calorimeter. Heat Capacity of some Polyphenyls at *T* = 298.15 K. *J. Chem. Thermodyn.* **2011**, *43*, 1818-1823.
2. Suurkuusk, J.; Wadsö, I. Design and Testing of an Improved Precise Drop Calorimeter for the Measurement of the Heat Capacity of Small Samples. *J. Chem. Thermodyn.* **1974**, *6*, 667-679.
3. Sabbah, R.; Xu-Wu, A.; Chickos, J. S.; Planas Leitão, M. L.; Roux, M. V.; Torres, L. A. Reference Materials for Calorimetry and Differential Thermal Analysis. *Thermochim. Acta* **1999**, *331*, 93-208.
4. Blanquart, G.; Pitsch, H. Thermochemical Properties of Polycyclic Aromatic Hydrocarbons (PAH) from G3MP2B3 Calculations. *J. Phys. Chem. A* **2007**, *111*, 6510-6520.
5. Della Gatta, G.; Richardson, M. J.; Sarge, S. M.; Stølen, S. Standards, Calibration, and Guidelines in Microcalorimetry. Part 2. Calibration Standards for Differential Scanning Calorimetry (IUPAC Technical Report). *Pure Appl. Chem.* **2006**, *78*, 1455-1476.
6. Domalski, E. S.; Hearing, E. D. Heat Capacities and Entropies of Organic Compounds in the Condensed Phase. Volume III. *J. Phys. Chem. Ref. Data* **1996**, *25*, 1-525.
7. Roux, M. V.; Temprado, M.; Chickos, J. S.; Nagano, Y. Critically Evaluated Thermochemical Properties of Polycyclic Aromatic Hydrocarbons. *J. Phys. Chem. Ref. Data* **2008**, *37*, 1855-1996.
8. Chickos, J. S. A Protocol for Correcting Experimental Fusion Enthalpies to 298.15 K and it's Application in Indirect Measurements of Sublimation Enthalpy at 298.15 K. *Thermochim. Acta* **1998**, *313*, 19-26.
9. Santos, L. M. N. B. F.; Lima, L. M. S. S.; Lima, C. F. R. A. C.; Magalhães, F. D.; Torres, M. C.; Schröder, B.; Ribeiro da Silva, M. A. V. New Knudsen Effusion Apparatus with Simultaneous Gravimetric and Quartz Crystal Microbalance Mass Loss Detection. *J. Chem. Thermodyn.* **2011**, *43*, 834-843.
10. Ribeiro da Silva, M. A. V.; Monte, M. J. S.; Santos, L. M. N. B. F. The Design, Construction, and Testing of a New Knudsen Effusion Apparatus. *J. Chem. Thermodyn.* **2006**, *38*, 778-787.
11. Barforoush, M. M.; Naderi, S.; Ghanbarpour, A. R.; Tehrani, A. A.; Khavasi, H. R. 2-Phenyl-1H-Imidazole. *Acta Cryst.* **2011**, *E67*, o3248.
12. Staples, R. J.; Sonderegger, L. H. Crystal Structure of 4-Phenylimidazole, $C_9H_8N_2$. *Z. Kristallogr.* **2001**, *NCS 216*, 313-314.
13. Stibrany, R. T.; Potenza, J. A.; Schugar, H. J. CCDC 172750: Experimental Crystal Structure Determination, **2002**, DOI: 10.5517/cc5srl8.
14. Lynch, D. E. CCDC 720571: Experimental Crystal Structure Determination, **2010**, DOI: 10.5517/ccs5t7z.
